# Intersystem Crossing Outcompetes Triplet-Pair Separation from ^1^(TT) below 270 K in Anthradithiophene Films

**DOI:** 10.1021/jacs.5c00001

**Published:** 2025-07-30

**Authors:** Eman M. Bu Ali, Arnau Bertran, Gabriel Moise, Shuangqing Wang, Rachel C. Kilbride, John E. Anthony, Claudia E. Tait, Jenny Clark

**Affiliations:** † School of Mathematical and Physical Sciences, 7315 The University of Sheffield, Sheffield S3 7RH, U.K.; ‡ Department of Physics, College of Science, King Faisal University, Hofuf, Al-Hassa 31982, Saudi Arabia; § Department of Chemistry, 6396 University of Oxford, Oxford OX1 3QR, U.K.; ∥ Department of Chemistry and Biochemistry, University of California, San Diego, La Jolla, California 92093, United States; ⊥ Department of Chemistry, 4530 University of Kentucky, Lexington, Kentucky 40511, United States

## Abstract

Singlet fission (SF) and triplet–triplet annihilation (TTA) are processes which may be exploited to boost the efficiency of solar energy technology. Despite being studied since the late 1960s, the mechanism of singlet fission is still not fully understood. This is partly because the main technique used to study singlet fission, optical or visible/near-IR transient absorption spectroscopy, cannot distinguish between the strongly coupled triplet-pair state ^1^(TT), weakly interacting triplet pairs (T..T), and independent triplet states T_1_ + T_1_. To solve this problem, we combine transient optical spectroscopy performed as a function of magnetic field and transient electron spin resonance (ESR) spectroscopy to probe the different steps involved in the singlet fission mechanism. By using transient photoluminescence spectroscopy performed as a function of magnetic field to selectively probe the second step of singlet fission: ^1^(TT) ⇌ (T..T), we show that in a well-studied model system, anthradithiophene (diF-TES-ADT), this step is highly temperature-dependent, even though the first step, ^1^S → ^1^(TT), is not. Transient ESR measurements confirm the absence of singlet fission at temperatures between 40 and 250 K for this system, with clear signatures of triplets generated by intersystem crossing and evidence for decay by triplet–triplet annihilation, further supported by magnetic field effect measurements. We conclude that in polycrystalline diF-TES-ADT, intersystem crossing outcompetes triplet hopping at temperatures below 270 K, enabling direct intersystem crossing from the bound triplet pair ^1^(TT) to an independent triplet state T_1_ localized on a single chromophore. The generated triplets can re-encounter and decay through triplet–triplet annihilation.

## Graphical abstract

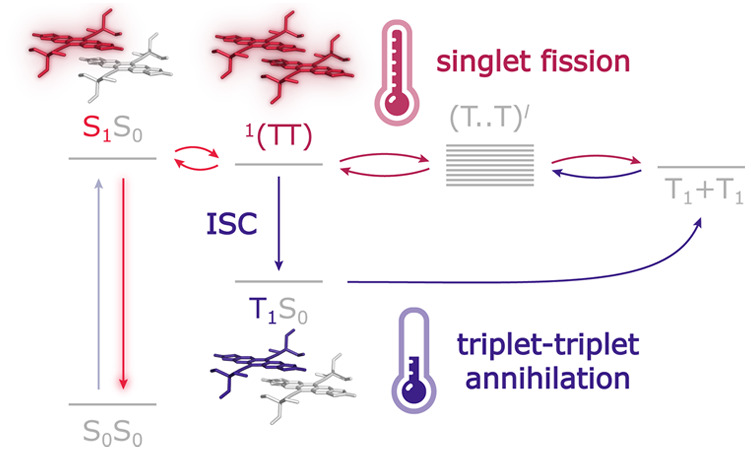

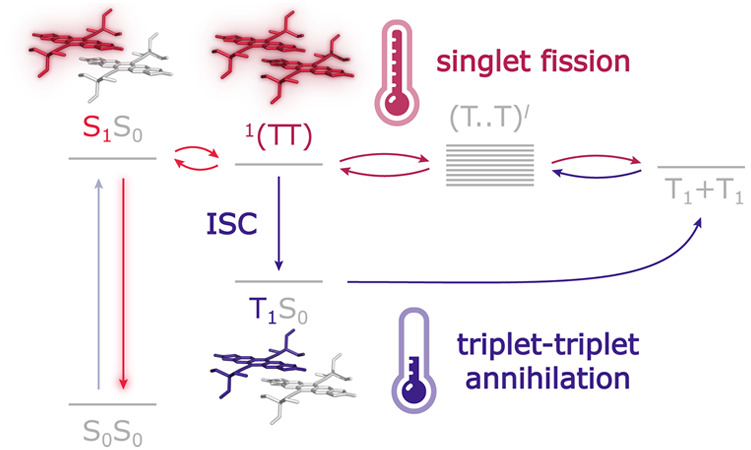

## Introduction

Singlet fission (SF) involves conversion of a high-energy photoexcited singlet exciton into a pair of lower energy triplet excitons.^1,2^ This multiexciton generation process has been studied over the past decade primarily because of its promise to improve solar cell efficiency through carrier multiplication,^1,3–6^ as a high-energy photon can generate two electron–hole pairs, reducing losses due to thermalization.^7,8^ Triplet–triplet annihilation (TTA) is the inverse process, in which a pair of low-energy triplet excitons are converted to a single high-energy singlet exciton.^9^ This process has been implicated in improving the performance of organic light emitting diodes (OLEDs),^10,11^ solar photovoltaics,^12,13^ biomedical applications^14^ including targeted drug delivery and optogenetics^15,16^ and three-dimensional (3D) printing.^17^

As shown schematically in Figure 1, the commonly accepted scheme of singlet fission is that a photoexcited singlet state S_1_ and a ground state singlet state S_0_ form a triplet-pair state, initially in an overall singlet configuration, known as ^1^(TT).^18–22^ Subsequently, this intermediate triplet pair separates to form a weakly bound triplet-pair state (T..T), and eventually two uncoupled triplet excitons. This is represented as S_1_S_0_ ⇌ ^1^(TT) ⇌ (T..T) ⇌ T_1_ + T_1_.^20,23^ Triplet–triplet annihilation is the inverse process, starting with independent triplets and resulting in an emissive singlet state.

**Figure 1:**
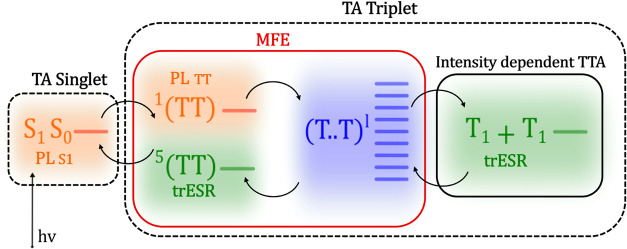
Schematic illustration of the different steps involved in the singlet fission process. The diagram illustrates the processes leading from the excited singlet state S_1_ to two triplet states T_1_ + T_1_ and the techniques used to probe them: the presence of S_1_ and triplet states after photoexcitation is detectable by transient absorption spectroscopy (dashed black lines); the presence and dynamics of S_1_ and ^1^(TT) can be detected by fluorescence spectroscopy (orange shades); ^5^(TT) and T_1_ + T_1_ are observable via trESR (green shades); the formation or recombination of (T..T) can be monitored through magnetic field effects (MFE) on photoluminescence (red line); processes that depend on exciton density, such as TTA, can be probed through intensity-dependent optical and ESR measurements (solid black line).

Understanding the nature of the intermediate triplet-pair states, and their fate, is key to fully exploiting singlet fission or triplet–triplet annihilation.^6,24–27^ The triplet-pair spin Hamiltonian that governs the nature of the triplet-pair intermediates ^1^(TT), ^5^(TT) and (T..T) in Figure 1 can be expressed in terms of the spin operators **Ŝ**_*i*_ on sites *A* and *B*

$$Ĥ=\underset{i=A,B}{\sum }\left[g_{i}\mu_{B}B\cdot {Ŝ}_{i}+D_{i}\left({Ŝ}_{i,z}^{2}-\frac{1}{3}{Ŝ}_{i}^{2}\right)+E_{i}\left({Ŝ}_{i,x}^{2}-{Ŝ}_{i,y}^{2}\right)\right]+J{Ŝ}_{A}\cdot {Ŝ}_{B}+{Ŝ}_{A}D_{\mathrm{AB}}{Ŝ}_{B}$$ 1

where *J* is the intertriplet exchange coupling (|*J*| ≫ |*D*| for ^1^(TT) or ^5^(TT) states and |*J*| ≪ |*D*| for (T..T) states, see below), *D*_*AB*_ indicates the intertriplet dipole coupling, **B** is the applied magnetic field strength and *D* and *E* (≫*D*_*AB*_) are the intratriplet zero-field splitting parameters. We note that the intertriplet dipolar term acts only as a weak perturbation. Other terms, such as the interactions between unpaired electrons and nuclei, are not included for simplicity.

Recent work^27–32^ has highlighted the importance of intertriplet exchange interactions *J* and their time dependence. Our scheme in Figure 1 reflects this by including both strongly exchange-coupled (^1^(TT)/^5^(TT)) and weakly exchange-coupled (T..T) triplet-pairs as separate states with interconversion between them. In many archetypal (hetero)­acene systems, such as diF-TES-ADT, pentacene and tetracene, the primary step of singlet fission is the formation of a strongly exchange-coupled triplet-pair state, ^1^(TT), where triplets within the pair reside on neighboring sites with orbital overlap.^7,20,33–35^

The strongly exchange-coupled triplet pairs are eigenstates of the triplet-pair spin Hamiltonian in eq 1 when |*J*| ≫ |*D*|. These states can in principle exist as pure spin singlet, triplet or quintet states: ^1^(TT), ^3^(TT), ^5^(TT) with total spin quantum number *S* = 0, 1, 2, respectively. Recent work shows that the singlet ^1^(TT) state, the primary product of singlet fission, can relax radiatively or nonradiatively to the singlet ground state.^36^ Alternatively, it can separate to form (T..T) or free triplet states, or it can interconvert to ^5^(TT) via singlet-quintet spin mixing mediated by the zero-field splitting interaction and affected by the strength and potential fluctuations in the exchange interaction.^37,38^ Evidence of quintet states is now well-established in exothermic singlet fission systems based on pentacene,^30,39–45^ and has been observed in TIPS-tetracene^29,46^ and a small number of other systems.^47–50^ However, quintets have not yet been observed in other archetypal endothermic singlet fission systems such as crystalline tetracene or diF-TES-ADT, despite the presence of strongly exchange-coupled ^1^(TT) states.^20,33^

We note that an equivalent description of “strongly exchange-coupled” triplet-pair states is of mixed triplet-pair/charge-transfer (CT) states, where the degree of CT character is directly related to the exchange interaction.^51^ The CT character of the ^1^(TT) state has recently been probed directly using time- and angle-resolved photoemission spectroscopy.^31^ This measurement shows that over time the CT character of the initially created ^1^(TT) state reduces as the triplets hop away from each other to form (T..T),^31^ as expected according to Wakasa et al.’s model of dynamic exchange^32^ and comparison between transient absorption and emission spectroscopy measurements.^33^

The (T..T)^*l*^ states formed in crystalline (hetero)­acene materials by triplet hopping from ^1^(TT) are the so-called ‘weakly coupled’ triplet-pair states. They make up the nine (*l* = 1, 2,···9) eigenstates of the spin Hamiltonian, eq 1, in the limit of weak exchange coupling, when |*J*| ≪ |*D*|. (T..T)^*l*^ are not spin eigenstates: spin is no longer a good quantum number and so the (T..T)^*l*^ states have mixed-spin character. This means that the rate of transition from ^1^(TT) to (T..T)^*l*^ is modulated by the number of (T..T)^*l*^ states with singlet character |*C*_*S*_^*l*^|^2^, where *C*_*S*_^*l*^ = ⟨^1^(TT)|(T..T)^*l*^⟩. The more (T..T)^*l*^ states have singlet character, the higher the rate of singlet fission. |*C*_*S*_^*l*^|^2^ depends on molecular orientation and applied magnetic field (through the Zeeman interaction, eq 1).^27,52–55^

Despite the depth of understanding of singlet fission in crystalline systems, and the consensus on the mechanism shown in Figure 1, several key questions remain to be resolved. A complete understanding of the singlet fission mechanism across different types of materials is complicated by the fact that different spectroscopic techniques selectively probe different parts of the process, see Figure 1—no single technique can be relied on to understand the entire photocycle. Transient absorption spectroscopy, for example (dashed black lines in Figure 1) probes excited state population. It can differentiate between S_1_ and triplets, but is unable to distinguish ^1^(TT), ^5^(TT), (T..T), and T_1_ + T_1_ since they usually exhibit comparable signatures.^56^ Photoluminescence (PL) spectroscopy (orange shading in Figure 1) offers information on S_1_ and ^1^(TT) populations,^20,55^ but can only indirectly monitor (T..T) or T_1_ + T_1_ populations.

Transient electron spin resonance (trESR) spectroscopy allows the measurement of states with nonzero overall electron spin, such as triplet and quintet states (green shading in Figure 1). The spin polarization pattern of the trESR spectra reveals the mechanism of formation of the detected photoinduced states and their role in decay processes.^57^

Light intensity-dependent optical and trESR measurements (solid black line in Figure 1) can provide additional information on processes that depend on exciton density, such as triplet–triplet annihilation.^20,54^

Finally, fluorescence-detected magnetic field effects (red line in Figure 1) are dominated by the formation or recombination of (T..T)^*l*^ through the |*C*_*S*_^*l*^|^2^ factors which represent the number of (T..T)^*l*^ states with singlet character (see above) and are governed by the spin Hamiltonian in eq 1.^28,58,59^ The dependence of the competition between singlet fission and ’prompt’ fluorescence on the parameters of the spin system can provide further insight into the singlet fission process. For identically oriented molecules, with parallel long axes (the typical case for (hetero)­acene crystals), at zero-field the number of (T..T)^*l*^ states possessing singlet character is three. At intermediate fields where *g*μ_B_ *B* ∼ *D* this increases to five before dropping to two at higher fields.^52^ This leads to the characteristic “singlet fission” magnetic field dependence observed originally in tetracene crystals:^60^ a drop in fluorescence as the field increases and spin mixing is favored, followed by an increase in fluorescence at higher fields, where fewer (T..T)^*l*^ states are able to mix with the singlet state. On the other hand, where triplet–triplet annihilation causes delayed fluorescence, for example when measuring at later times after excitation or in anthracene crystals where singlet fission is not energetically feasible, increased coupling of the (T..T)^*l*^ states to singlet states will give increased delayed fluorescence, hence the magnetic field dependence of the delayed fluorescence has the same shape as that of singlet fission, but with opposite sign.^58^

In this work, we study the temperature-, fluence-, and magnetic field-dependence of photoluminescence of a well-characterized anthradithiophene (diF-TES-ADT) singlet fission system.^20,33^ Previous studies based on transient absorption and photoluminescence spectroscopy have suggested that the first step of singlet fission, the generation of ^1^(TT), is temperature-independent in polycrystalline films of this material.^20,33^ However, as we show here, magnetic field-dependent photoluminescence spectroscopy reveals that the separation to form (T..T) is highly temperature-dependent in this material. TrESR measurements demonstrate the absence of singlet fission at temperatures below 250 K and no indication of quintet states, indicating population of triplet states by an intersystem crossing (ISC) process instead.

We explain these apparently contradictory observations by proposing that the biexcitonic ^1^(TT) state, formed at all temperatures and delocalized over two molecules, can itself undergo intersystem crossing to form a T_1_S_0_ state, with a triplet localized on only one molecule. While full singlet fission to produce (T..T) does not occur at low temperatures (below 270 K), the intersystem-crossing-generated triplets can nevertheless undergo thermally activated triplet–triplet annihilation over a wide range of temperatures.

## Results and Discussion

We selected 2,8-difluoro-5,11-bis­(triethylsilylethynyl) anthradithiophene (diF-TES-ADT, see molecular structure in Figure 2) as a model system to investigate the mechanism of singlet fission and triplet–triplet annihilation by measuring magnetic field effects (MFE) on photoluminescence.^61^ diF-TES-ADT is a well-characterized system^20,33,62,63^ with a simple brickwork crystalline structure and no apparent phase transition between 100 K and room temperature (RT).^64^ This material, furthermore, is air- and photostable, allowing for reproducible preparation of samples with the same properties.^65^

**Figure 2:**
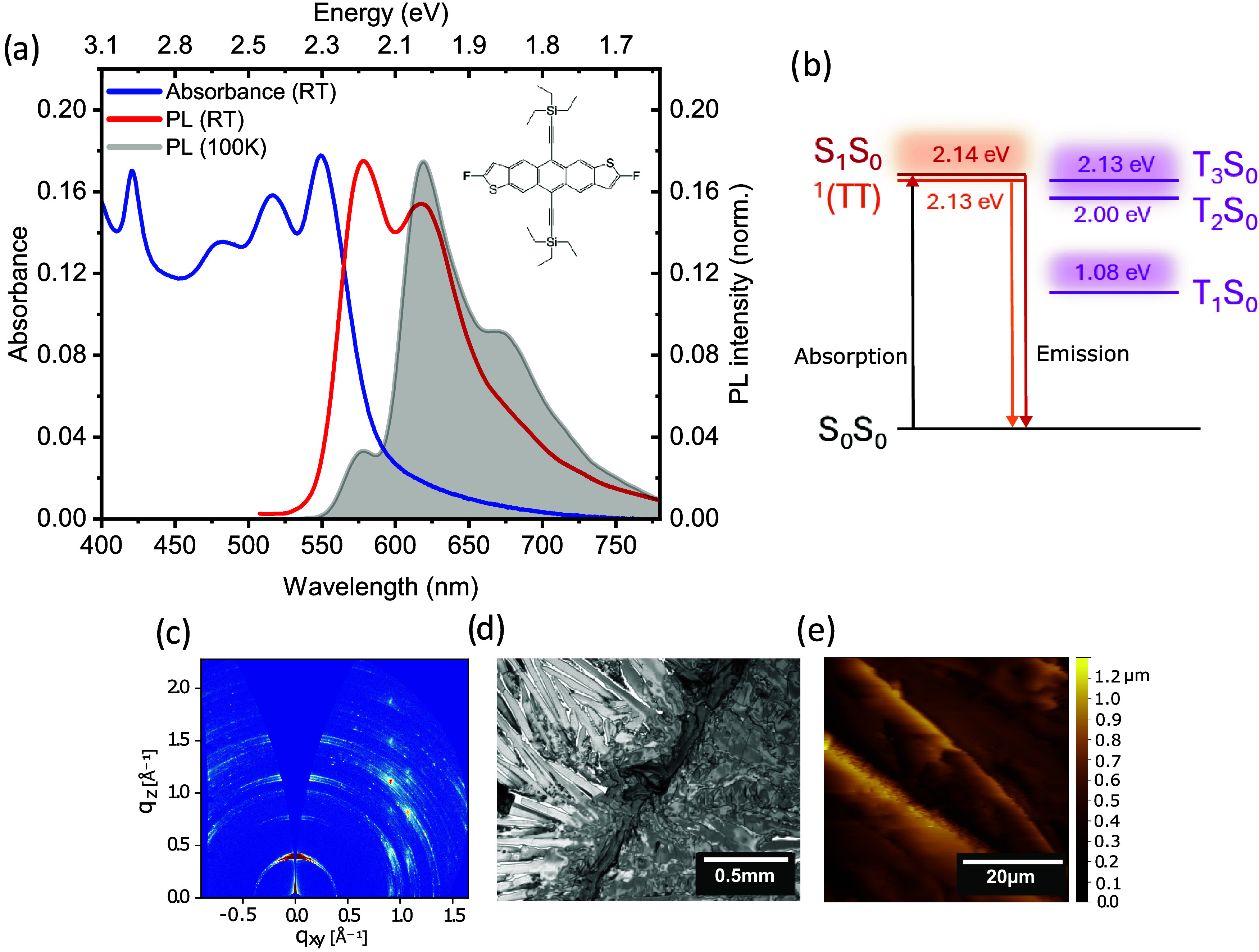
(a) diF-TES-ADT thin film steady-state room temperature absorption spectrum (blue) and photoluminescence (PL) spectra at room temperature (red) and 100 K (gray shaded). The chemical structure of diF-TES-ADT is shown in the inset. (b) Energy level diagram for diF-TES-ADT based on phosphorescence and transient absorption experiments in refs 33,67 . (c) GIWAXS pattern, (d) polarized microscope image, and (e) AFM scan of a diF-TES-ADT drop-cast film indicating highly crystalline domains. Scale bars are shown in the figure. Morphological characterization of the spin-coated films are presented in the Supporting Information, Figure S1a.

Absorption and photoluminescence spectra of diF-TES-ADT in thin films measured at room temperature (Figure 2a) display the vibronic progression associated with the transition between the electronic ground state S_0_ and the first excited state S_1_.^64,66^ However, when the temperature is reduced to 100 K, the emission spectrum exhibits a distinct peak that is displaced toward longer wavelengths compared to the RT spectrum. Over the same temperature range, the absorption spectrum narrows and redshifts slightly, but otherwise remains largely unchanged.^20,33^ The emission at 100 K has been assigned to a strongly coupled triplet-pair state ^1^(TT).^20,33^ Further temperature-dependent steady-state photoluminescence (PL) measurements demonstrate an increase of the strongly coupled triplet-pair state ^1^(TT) emission down to 100 K,^20,33^ as illustrated in Supporting Information Figure S4.

To characterize the morphology and microstructure of the diF-TES-ADT films used in this work, we used grazing incidence wide-angle X-ray scattering (GIWAXS), atomic force microscopy (AFM) and polarized microscopy (Figure 2). AFM and polarized microscopy measurements show that the drop-cast diF-TES-ADT film is composed of a micron-scale crystalline texture (Figure 2c,d). This is confirmed using GIWAXS measurements with the two-dimensional (2D) GIWAXS pattern consisting of several distinct scattering features indicating a highly crystalline film (Figure 2b). Further inspection of corresponding one-dimensional (1D) GIWAXS intensity profiles shows that the crystal structure is consistent with the previously reported brickwork packing with a predominantly edge-on lamellar motif (see Supporting Information Figure S2 for further details).

For a more detailed investigation of the steps of the singlet fission mechanism following ^1^(TT) formation, the impact of a magnetic field on the photoluminescence was evaluated by measuring PL spectra at magnetic field intensities ranging from 0 to 300 mT, and at delay times from 5 ns to 1 μs. The PL spectra were recorded while repeatedly changing the magnetic field strength in both upward and downward directions to ensure that the PL spectra obtained in both cases have the same shape and magnitude. The full measurement procedure is described in the Supporting Information.

Figure 3 displays the MFE data of a diF-TES-ADT drop-cast film at room temperature and 100 K. In Figure 3(a,c), the data is plotted as a function of magnetic field strength for a range of gate delay times. Our data at room temperature (Figure 3a) reproduces the results of earlier work by Bossanyi et al.:^20^ the prompt fluorescence intensity, from 5 to 20 ns, reflects singlet fission behavior, characterized by a decrease in ΔPL/PL (%) at lower magnetic fields and an increase at higher fields^60^ and the delayed fluorescence from 30 ns to 1 μs displays the inverted behavior characteristic of triplet–triplet annihilation.^58^ To more clearly visualize the temporal evolution, the same data is depicted in Figure 3b as a function of delay time for various magnetic field strengths, ranging from 0 to 300 mT. This graph shows that singlet fission is active over a time scale of 5 to 30 ns, whereas triplet–triplet annihilation dominates at long times and starts to outweigh the singlet-fission contribution beyond about 30 ns.

**Figure 3:**
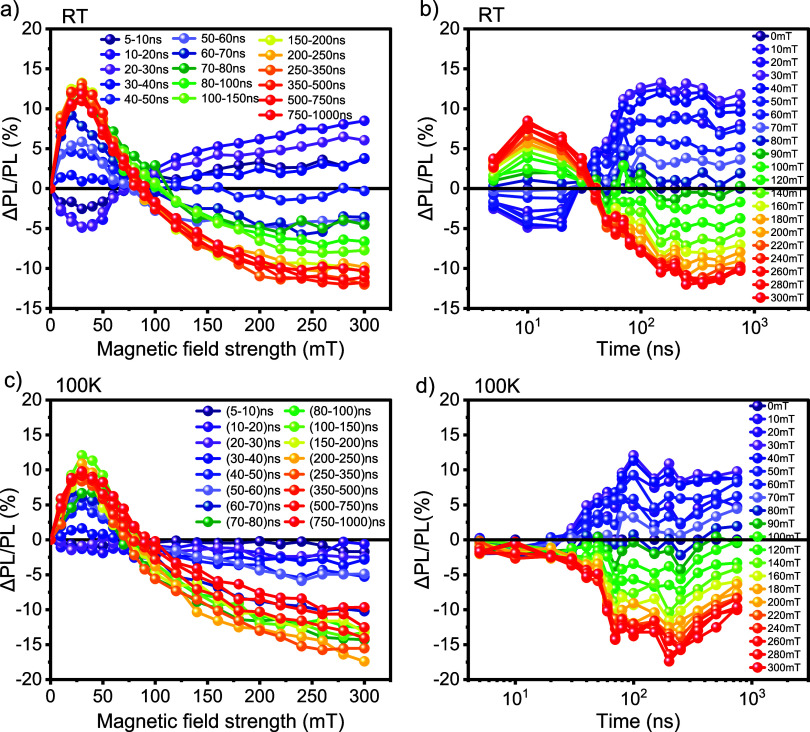
Magnetic field effects on photoluminescence for diF-TES-ADT drop-cast film measured at 532 nm across different delay times at room temperature and 100 K. ΔPL/PL (%) is reported as a function of (a, c) magnetic field strength (from 0 to 300 mT) and (b, d) delay time (from 5 ns to 1 μs).

We next investigated the temperature dependence of the MFE. Figure 3b shows the change in ΔPL/PL (%) as a function of magnetic field strength at 100 K, employing identical time intervals and magnetic field strengths as for the room temperature data discussed above. Surprisingly, upon reducing the temperature to 100 K, no MFE was observed between 5–20 ns suggesting that singlet fission does not occur at 100 K. However, the signature from triplet–triplet annihilation persisted with a similar magnitude and duration, from 30 ns to 1 μs, as observed at room temperature. This change in behavior is highlighted in Figure 3c, where the time-dependence of the magnetic field effect on the photoluminescence emphasizes the absence of singlet fission but, surprisingly, persistence of triplet–triplet annihilation.

In order to identify the temperature at which the singlet fission signature is no longer observable, we performed MFE measurements on a drop-cast diF-TES-ADT sample at a series of temperatures between 100 K and room temperature and the results are shown in Figure 4. At early delay times, 5–10 ns, the signature from singlet fission is only apparent at 270 K and room temperature (Figure 4a), suggesting an onset of singlet fission between 250 and 270 K (Figure 4b). On the other hand, the signature from triplet–triplet annihilation, shown for a delay time of 100–200 ns, is observed across the whole temperature range (Figure 4c). These results suggest that triplet–triplet annihilation is present independently from singlet fission.

**Figure 4:**
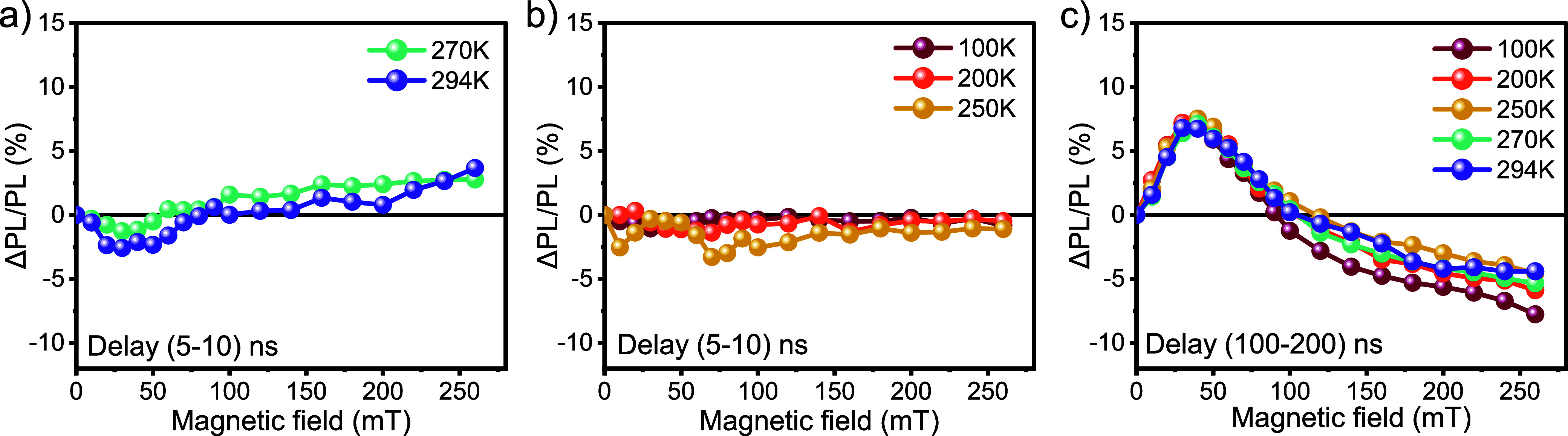
Temperature-dependent MFE of diF-TES-ADT drop-cast film at different delay times: (a, b) 5–10 ns at temperatures ranging from 270 K to RT, and from 100 to 250 K, respectively, (c) 100–200 ns across the temperature range from 100 K to RT.

The triplet–triplet annihilation process was probed further through fluence-dependent MFE measurements shown in Figure 5. Using a drop-cast diF-TES-ADT film at 100 K and a delay time of 100–250 ns, the magnetic field effect on photoluminescence was recorded while increasing the laser power from 11 μW to 2.3 mW. A noticeable decrease in the MFE is observed as the laser power increases. Similar behavior in a perylene/PtOEP system has been recently attributed to bimolecular triplet–triplet annihilation.^54^ At low excitation power, the probability that two triplets encounter and annihilate within their lifetime is low and at first increases quadratically with excitation intensity. At high excitation intensities, the triplet density becomes so high that most triplets annihilate within their excited state lifetime, giving a probability of triplet decay by triplet–triplet annihilation tending toward unity. In this regime, the delayed fluorescence intensity starts to depend linearly on excitation intensity. The strength of the magnetic field effect on the delayed fluorescence is determined by the relative magnitude of the first-order rate constant describing spin-independent triplet decay compared to the second-order rate constant of the spin-dependent triplet–triplet annihilation process and the triplet density.^54^ The delayed fluorescence is influenced most by an applied magnetic field at low excitation intensities and therefore low triplet densities. At high excitation intensities, the impact of the magnetic field on singlet formation is reduced as a larger proportion of triplet pairs undergoes fusion before experiencing significant spin evolution under the applied magnetic field. The fluence-dependent MFE experiments therefore confirm that our data at 100 K can be explained by a bimolecular triplet–triplet annihilation process.

**Figure 5:**
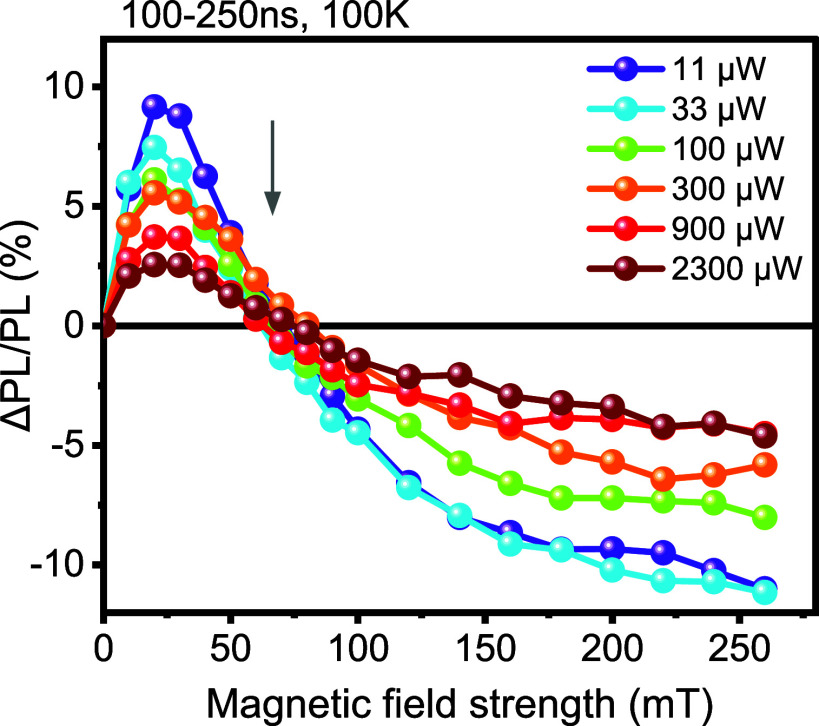
Fluence-dependent magnetic field effect data of diF-TES-ADT drop-cast film measured at 532 nm at 100 K and a delay time of 100–250 ns. The magnetic field effect on photoluminescence was recorded while increasing the laser power from 11 μW to 2.3 mW.

To provide further insights into the apparent absence of singlet fission at low temperatures and into the origin of the triplet states that are observed to be involved in TTA, the magnetic field effect measurements were complemented by transient electron spin resonance (trESR). trESR spectroscopy directly probes the nature and dynamics of photoinduced spin states with *S* > 0. Their mechanism of formation is encoded in the spin polarization pattern of the trESR spectra, arising from non-equilibrium populations of the spin sublevels.^57^ Triplet and quintet states formed by singlet fission are usually characterized by an initial spin polarization pattern resulting from selective population of the *m*_*S*_ = 0 sublevel due to spin conservation during the singlet fission process. The initial, ESR-silent, strongly coupled triplet pair ^1^(TT) is generated in a spin-zero state from the excited singlet state precursor and results in population of the eigenstates of the coupled pair of triplets, and the resulting separated triplet states, with probabilities determined by their singlet content.^29,30^ The spectral signatures of triplets populated by intersystem crossing (ISC), on the other hand, are determined by spin-selective population of the zero-field spin sublevels driven by spin–orbit coupling, resulting in clearly distinct spin polarization patterns.^57^

Figure 6 shows the results of transient ESR measurements performed on drop-cast diF-TES-ADT films at temperatures between 40 and 250 K. The full evolution of the ESR spectrum as a function of time after photoexcitation for each temperature is shown on the left (red = emissive, blue = absorptive transition), and transients extracted at selected field positions as well as spectra extracted at early times after photoexcitation are shown in the middle and on the right.

**Figure 6:**
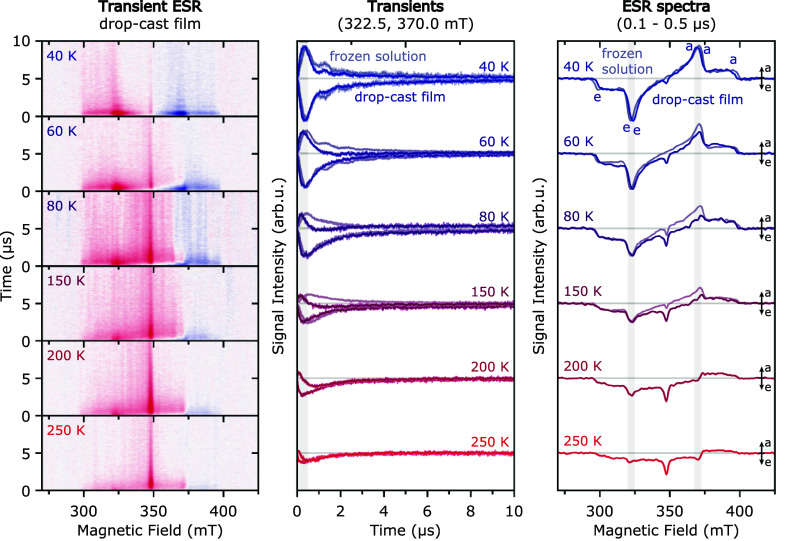
Transient ESR measurements on diF-TES-ADT drop-cast films at a series of temperatures. Time-dependent evolution of the ESR spectra as a function of time after laser excitation (*left*, red = emissive, blue = absorptive), transients extracted at the field positions corresponding to the *X* canonical field positions (322.5 mT and 370.0 mT, *center*) and spectra extracted at early times after laser excitation (0.1–0.5 μs, *right*). For temperatures up to 150 K, the scaled transients and ESR spectra recorded on frozen solutions are displayed in the background. See SI for experimental details.

At 40 K, the ESR spectrum recorded for diF-TES-ADT in drop-cast films exhibits a triplet state ESR spectrum with an eeeaaa (e = emissive, a = absorptive) spin polarization pattern. The spectrum is characterized by zero-field splitting parameters |*D*| = 1370 ± 5 MHz and |*E*| = 50 ± 5 MHz, in good agreement with previously reported values from optically detected magnetic resonance (ODMR).^33^ The spin polarization pattern corresponds to relative populations of *p*_*X*_: *p*_*Y*_: *p*_*Z*_ = 0.45:0.36:0.19 across the zero-field spin sublevels of the triplet state (for *D* > 0, *E* < 0 in analogy to triplet states on other polyacenes, and supported by DFT calculations, see SI, Figure S11 for details). This is characteristic of triplet states on polyacenes generated by intersystem crossing (ISC), with selective population of the T_*X*_ and T_*Y*_ sublevels, associated with the two in-plane symmetry axes, by vibrational spin–orbit coupling.^68,69^ This spin polarization pattern is clearly distinct from the aeeaae pattern expected for triplet states generated through singlet fission with selective population of the high-field T_0_ sublevel independent of orientation (see Figure S10 in the SI). Therefore, we conclude that the triplet state observed for diF-TES-ADT at low temperatures is generated by ISC, as further confirmed by the excellent agreement between the spin polarization pattern of the ESR spectra recorded for the drop-cast film and for diF-TES-ADT in dilute frozen solution (|*D*|= 1420 ± 5 MHz, |*E*| = 32 ± 5 MHz, *p*_*X*_: *p*_*Y*_: *p*_*Z*_ = 0.45:0.36:0.19[Fn fn1]).

As the temperature is increased from 40 to 250 K, the transient ESR spectra recorded for the drop-cast films at 0.1–0.5 μs after photoexcitation change from a symmetric eeeaaa spin polarization pattern to a mostly emissive spin polarization. At each temperature, the spin polarization evolves from a symmetric eeeaaa pattern at very short times after laser excitation to a mostly emissive spin polarization at longer times. The net emissive contribution becomes more evident and starts contributing at earlier times as the temperature increases. Overall, the signal intensity of the observed triplet states decreases for increasing temperatures. The observation of a net emissive polarization building up over time is unique to the drop-cast films, while a symmetric polarization pattern still persists for the frozen solution spectra in the probed temperature range (40 to 150 K, below the freezing point of toluene at 178 K), as evident from the comparison of transients and ESR spectra in Figure 6 (see also Figure S9 in the SI for the full data set recorded in frozen solution).

A narrow emissive feature at 348 mT (*g* ≈ 2.004) also becomes more prominent for increasing temperatures. Similar features have been observed in other singlet fission materials^41,70^ and could originate either from motionally averaged highly mobile triplet states or from radical pairs formed by photoinduced charge transfer. In diF-TES-ADT, the delayed rise of this contribution compared to that of the triplet state signal (see Figure S12 in the SI for details), the narrow spectral width and the emissive polarization, lead us to speculate that this contribution may be due to a spin-correlated radical pair formed by charge separation after formation of the triplet state by ISC, and probably in conjunction with the triplet–triplet annihilation process, given that the relative weight of this contribution increases with the increased emissive triplet state polarization resulting from spin-selective annihilation. The emissive polarization is inherited from the spin-polarized triplet precursor.^71^ We did not observe any contribution from strongly coupled triplet pairs forming a quintet state for diF-TES-ADT at any of the investigated temperatures (see Figure S10 in the SI for a simulation of the expected spectrum), in contrast to what has been previously observed for exothermic singlet-fission materials.^29,30,39^

A delayed net emissive triplet state spin polarization has previously been proposed to result from triplet–triplet annihilation in antiferromagnetically coupled triplet pairs^72^ and first observed experimentally in anthracene-tetracyanobenzene and phenazine-tetracyano-quinodimethane molecular crystals.^73^ The origin of the delayed net emissive spin polarization can be traced back to the spin selectivity of triplet–triplet annihilation. The encountering triplets can form pairs with total spin 0, 1 or 2, but annihilation is selective for encounter pairs with overall spin 0 due to spin conservation. The remaining coupled triplet pairs with overall spin 1 or 2 undergo spin mixing driven by spin–spin interactions and, combined with the selective annihilation of any pairs with overall spin 0, this leads to the build-up of a spin polarization that is inherited by the individual triplet states after separation.^72^ Generation of this spin polarization requires mobile triplet states, which can encounter within the material and separate again, and the presence of spin–spin interactions in the coupled triplet pairs surviving the encounter. The absence of a net emissive spin polarization at low temperatures (≤ca. 40 K), where triplet exciton diffusion is frozen out, and the increase of this contribution for increasing temperatures, and therefore increasing triplet exciton mobility, supports interpretation of the evolution of spin polarization observed for diF-TES-ADT films in terms of triplet–triplet annihilation.

The presence of triplet–triplet annihilation is further confirmed by fluence-dependent trESR measurements performed at 100 K and shown in Figure 7. An increasing contribution of the delayed net emissive polarization, relative to the initial eeeaaa polarization pattern, is observed for higher laser fluences, indicating dependence of this polarization on the number of triplet excitons, as expected for polarization originating from a bimolecular triplet–triplet annihilation process.^73^

**Figure 7:**
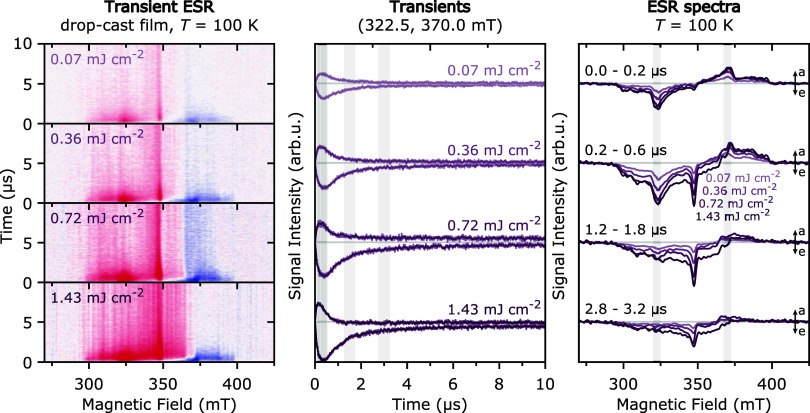
Transient ESR measurements on diF-TES-ADT drop-cast films at 100 K for different laser fluences. Time-dependent evolution of the ESR spectra as a function of time after laser excitation (*left*, red = emissive, blue = absorptive), transients extracted at the field positions corresponding to the *X* canonical field positions (322.5 and 370.0 mT, *center*) and comparison of spectra extracted at different times after laser excitation (*right*). See SI for experimental details.

The evolution of spin polarization of the triplet state spectra observed for the diF-TES-ADT films at different temperatures and laser fluences can be modeled using a kinetic scheme originally proposed by Corvaja et al.^73^ and including the spin-selective unimolecular decay of the triplet sublevel populations, spin relaxation, and, importantly, the bimolecular decay by triplet–triplet annihilation (see SI Section 6.5). The resulting simulations of spectra extracted at short times after photoexcitation are compared to the experimental results in Figure 8. To minimize the number of fitting parameters, the rate constants for spin-selective triplet decay and relaxation were first extracted from simulations of the frozen solution trESR spectra. Simulations for the drop-cast films were then performed by only varying the rate constants for triplet–triplet annihilation. As can be seen from the comparison of the experimental results with simulations in Figure 8, the kinetic scheme of Corvaja et al. captures all the important features of the trESR spectra for the different experimental conditions. The simulations confirm that triplet–triplet annihilation plays an increasing role at higher temperatures and higher excitation intensities.

**Figure 8:**
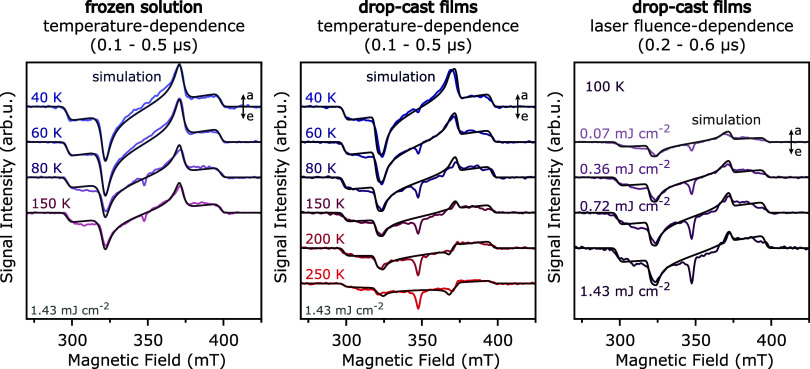
Comparison of experimental transient ESR spectra with simulations: results of measurements on diF-TES-ADT frozen solutions and drop-cast films at a series of temperatures and at different laser fluences are modeled considering spin-selective decay kinetics, relaxation processes and the effect of triplet–triplet annihilation. See SI for simulation parameters and details of the simulation procedure.

The absence of singlet fission-generated triplet states in trESR measurements and no evidence for the occurrence of singlet fission in the MFE data recorded below 270 K (Figure 4), in combination with the observation of features originating from triplet–triplet annihilation in both MFE and trESR measurements, indicate that bimolecular triplet–triplet annihilation dominates at low temperatures. TrESR further demonstrates that the triplets which undergo bimolecular triplet–triplet annihilation are initially generated by intersystem crossing. However, transient absorption and emission spectroscopy^20,33^ show that ^1^(TT) is formed with a ≈200 ps time-constant, independent of temperature.^20,33^ Therefore, spin-forbidden intersystem crossing from S_1_ is unlikely to compete with the initial ^1^(TT) formation, and this is supported by the lack of long-lived triplets in transient absorption spectroscopy at room temperature.^33^ We therefore conclude that the ISC-generated triplets observed in trESR originate from ^1^(TT) via intersystem crossing. At room temperature, we hypothesize that ^1^(TT) separation to form (T..T) is competitive with intersystem crossing and full singlet fission occurs according to S_1_S_0_ → ^1^(TT) →(T..T), as observed in the MFE measurements. At temperatures below 270 K, however, intersystem crossing outcompetes triplet-pair separation and free triplets are mainly generated by intersystem crossing.

^1^(TT) → T_1_S_0_ is not unprecedented, as intersystem crossing is known to occur in carotenoids from the lowest-lying S_1_ state^74^ and, in these molecules, S_1_ can be described as an intramolecular ^1^(TT) state.^36^ In addition, the near-degeneracy between ^1^(TT) and high-lying triplet states T_2_ and T_3_ in diF-TES-ADT should favor intersystem crossing. We can estimate these triplet state energies from previous studies: from phosphorescence spectra^33^ *E*_T_1__ = 1.08 eV, and from transient absorption spectra,^67^ the vertical triplet energies (at the T_1_ geometry) are *E*_T_2__ = 2.20 eV and *E*_T_3__ = 2.33 eV. Assuming relaxation along the triplet potential energy surface to be on the order of 200 meV,^75,76^ we put the relaxed triplet energies at *E*_T_2__ = 2.00 eV, *E*_T_3__ = 2.13 eV. These triplets are almost degenerate with ^1^(TT)­( *E* ^1^(TT) = 2.13 eV).^33^ It is therefore reasonable that intersystem crossing can occur from ^1^(TT) to T_2_S_0_ and/or T_3_S_0_ followed by rapid internal conversion to T_1_S_0_, as shown in the energy level diagram in Figure 2b.

We note that it is possible that T_1_ is generated from ^1^(TT) via a different mechanism, where ^1^(TT) converts first to ^3^(TT) before internally converting to T_1_. However, since the ^3^(TT) states are antisymmetric relative to interchange of the two partners and the ^1^(TT) and ^5^(TT) are symmetric, the ^1^(TT) → ^3^(TT) conversion is less efficient than ^1^(TT) → ^5^(TT). Therefore, we would expect that if ^3^(TT) were generated, ^5^(TT) should also be generated, and we see no evidence of quintets in the trESR data.

To test our hypothesis, we simulated the MFE data at both RT and 100 K using a model based on the modified Merrifield kinetic model described in ref 20. This modified Merrifield model has been shown to correctly simulate the predicted room temperature MFE data at both early and late delay times for diF-TES-ADT films.^20^ Here, we further modified the model to include temperature-dependent triplet hopping and intersystem crossing from ^1^(TT).

Figure 9 f our kinetic model. The associated rate equations are

$$\begin{array}{l} \frac{d\left[S_{1}\right]}{dt}=-\left(k_{\mathrm{sf}}+k_{\mathrm{snr}}\right)\left[S_{1}\right]+k_{-\mathrm{sf}}{[}^{1}\left(\mathrm{TT}\right)]\\ \frac{d{[}^{1}(\mathrm{TT})]}{dt}=k_{\mathrm{sf}}\left[S_{1}\right]-\left(k_{-\mathrm{sf}}+k_{\mathrm{ISC}}+k_{{hop}^{*}}\overset{9}{\underset{l=1}{\sum }}|C_{S}^{l}{|}^{2}+k_{{ttnr}^{*}}\right){[}^{1}\left(\mathrm{TT}\right)]+k_{-\mathrm{hop}}\overset{9}{\underset{l=1}{\sum }}|C_{S}^{l}{|}^{2}[\left(T..T{)}^{l}\right]\\ \frac{d[\left(T..T{)}^{l}\right]}{dt}=k_{{hop}^{*}}|C_{S}^{l}{|}^{2}{[}^{1}\left(\mathrm{TT}\right)]-\left(k_{-\mathrm{hop}}|C_{S}^{l}{|}^{2}+k_{\mathrm{hop}2}+k_{\mathrm{tnr}}+k_{\mathrm{relax}}\right)[\left(T..T{)}^{l}\right]+\frac{1}{9}k_{\mathrm{tta}}{\left[T_{1}\right]}^{2}+\frac{1}{8}k_{\mathrm{relax}}\underset{j\neq l}{\sum }[\left(T..T{)}^{j}\right]\\ \frac{d\left[T_{1}\right]}{dt}=\left(k_{\mathrm{tnr}}+2k_{\mathrm{hop}2}\right)\overset{9}{\underset{l=1}{\sum }}\left[\left(T..T{)}^{l}\right]+k_{\mathrm{ISC}}{[}^{1}\left(\mathrm{TT}\right)\right]-2k_{\mathrm{tta}}{\left[T_{1}\right]}^{2}-k_{\mathrm{tnr}}\left[T_{1}\right] \end{array}$$

The rate constants used to model the magnetic field effects were obtained by fitting time-resolved photoluminescence measurements on a diF-TES-ADT film as a function of temperature and laser fluence, as described in ref 20. In that work, we also simulated the magnetic field effects using only rates obtained from time-resolved spectroscopy. Here, having modified the rate model from our previous work to include temperature-dependent triplet hopping and intersystem crossing terms, we modified the original rates only slightly to reduce the number of fitting parameters to a minimum (see Supporting Information for more details). For example, while in ref 20, *k*_ttnr_ was used to describe both ISC and the nonradiative decay of ^1^(TT), here we explicitly include *k*_ISC_ and denote the rate constant of nonradiative processes as *k*_ttnr^*^_, to distinguish it from the overall rate used previously. In addition, we modified *k*_hop_ to include a temperature dependence: $k_{{\mathrm{hop}}^{*}}=k_{{\mathrm{hop}}^{{}^{\circ}}}e^{\left(-\Delta E/k_{B}T\right)}$ , where *k*_hop^°^_ is calculated based on *k*_hop_ at RT from ref 20, *k*_B_ is the Boltzmann constant, and Δ*E* is the activation energy for triplet-pair separation (Δ*E* = 20 meV according to ref 33).

**Figure 9:**
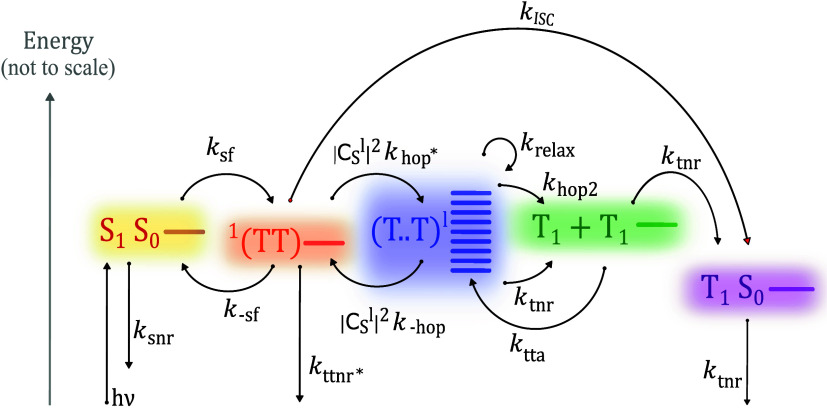
Updated kinetic scheme involving singlet fission at temperatures ranging from 270 K to RT and intersystem crossing at temperatures below 270 K. The rates used in our model are marked in the figure. Here, *k*_snr_, *k*_ttnr*_, and *k*_tnr_ include both radiative and nonradiative decay to the ground state. The relative energies are *not* to scale (separation between T..T levels is on the order of 1–10 μeV, and exchange energy 2*J* between S_0_S_1_/S_0_T_1_ is on the order of 1 eV, while the difference between ^1^(TT) and T_1_ + T_1_ is ∼30 meV).

As presented in Figure 10, we find that the simulation and the data of the MFE at RT (Figure 10a,b) and 100 K (Figure 10c,d) are in reasonably good agreement in terms of the shape, intensity, and zero-crossing over the entire time range. There are some discrepancies in the dynamics, which are due to the simplicity of the model, but overall the physics of the system is well represented by the model.

**Figure 10:**
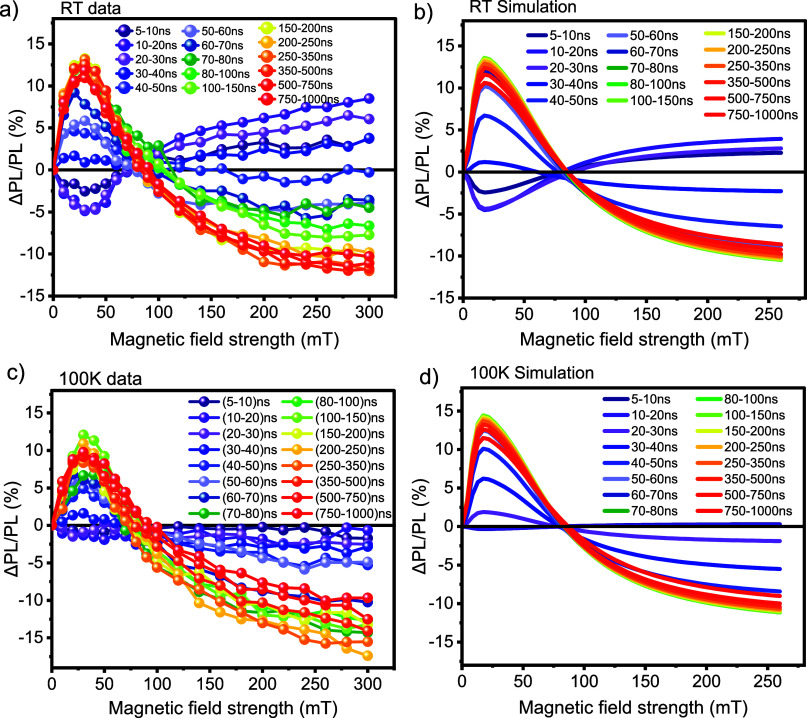
Comparison of experimental results and simulations of MFEs on the photoluminescence of the diF-TES-ADT drop-cast film measured at 532 nm across different delay times, from 5 ns to 1 μs, at (a, b) room temperature and (c, d) 100 K, respectively. The simulation and the data of the MFE at RT and 100 K are in reasonably good agreement in terms of the shape, intensity, and zero-crossing over the entire time range.

In addition, Figure 11 shows simulations of the power-dependent MFE data reported in Figure 5. For this simulation, the reported laser power, measured in μW, had to be converted to the exciton density, measured in cm^–3^. However, the potential excitation densities vary by approximately 3 orders of magnitude due to the non-uniform thickness of the drop-cast film. Accurately determining the excitation densities for this specific sample is somewhat challenging. Consequently, we use the thin film excitation densities in our simulation since we possess precise measurements of the films thickness. Details of the fluence dependence simulation and exciton density calculation are included in the Supporting Information.

**Figure 11:**
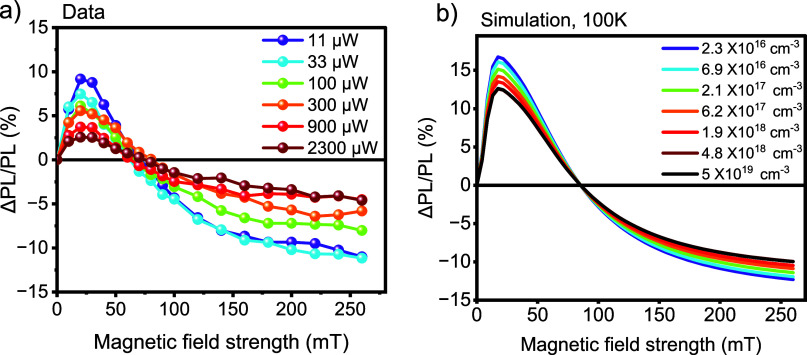
Comparison of (a) experimental results and (b) simulation of the fluence-dependent magnetic field effect of diF-TES-ADT drop-cast film measured at 523 nm at 100 K. The simulation shows the drop in the TTA-MFE as the exciton density rises and broadly shows good agreement with the experimental MFE behavior.

The simulation in Figure 11b reproduces the drop in the TTA-MFE as the exciton density rises and broadly shows good agreement with the experimental MFE behavior of Figure 11a.

In conclusion, in this study we showed that, despite a temperature-independent formation of strongly exchange-coupled ^1^(TT) in crystalline diF-TES-ADT films, demonstrated in earlier work,^20,33^ the formation of weakly coupled (T..T) is highly temperature-dependent. Fluorescence-detected magnetic field effects, for example, showed no signature of singlet fission below 270 K and transient ESR showed no signatures of quintet states or singlet fission-generated triplets at any temperature between 40 and 250 K. Instead, our results suggest direct intersystem crossing (ISC) from bound triplet pairs to individual triplet states, ^1^(TT) → T_1_S_0_, takes place at low temperatures, whereas singlet fission only becomes dominant at higher temperatures. These findings, supported by magnetic field effect and trESR experiments, reveal an additional decay pathway of the biexcitonic ^1^(TT) state through ISC and therefore an additional variable to consider when designing singlet fission materials for quantum or solar applications.

In organic systems without heavy atoms, ISC is mostly driven by vibronic spin–orbit coupling.^77^ A competition between ISC from S_1_ and singlet fission has previously been observed in crystalline tetracene^78^ and other systems,^50,79–81^ where ISC was enhanced by small energy gaps or near degeneracy of S_1_ and high energy triplet states or spin–orbit charge-transfer ISC.^81^ This work thus adds to an increasing amount of evidence that ISC can outcompete singlet fission, in the case of diF-TES-ADT, even following the initial step of singlet fission forming the ^1^(TT) state, through a potentially important additional loss pathway.

Even though singlet fission is suppressed at low temperatures in diF-TES-ADT, triplet–triplet annihilation of the ISC-born triplet states remains allowed down to cryogenic temperatures, where decreased triplet mobility starts to prevent encounters. Generation of the ^1^(TT) state not only by singlet fission but also by bimolecular triplet–triplet annihilation, as observed in previous optical studies,^20^ allows comparison of the role and dynamics of this state in the two multiexciton processes. We find that, in diF-TES-ADT films, bimolecular triplet–triplet annihilation does not populate quintet states efficiently enough, or the formed states are not long-lived enough, to be observed with trESR, as opposed to significant evidence for contribution of these states to the singlet fission mechanism in several other materials.^29,30,39–50^ This finding could indicate stabilization of ^1^(TT) with respect to ^5^(TT), as expected from configuration interaction arguments^51^ and may have implications for triplet–triplet annihilation up-conversion.

Finally, our study further highlights the importance of combining a range of different optical and magnetic resonance spectroscopic techniques to obtain a full picture of the photophysical processes in materials for singlet fission and triplet–triplet annihilation. Only by combining the ability to identify formation of the ^1^(TT) state by photoluminescence spectroscopy, the unequivocal assignment of the formation mechanism of the observed independent triplet states to ISC based on the spin polarization pattern in trESR spectra and the evidence for bimolecular triplet–triplet annihilation from both temperature- and fluence-dependent magnetic-field-dependent photoluminescence and trESR experiments, we were able to fully unravel the photophysics of the diF-TES-ADT system.

## Supplementary Material



## Data Availability

Data from this manuscript will be available on the Sheffield Repository (ORDA) with a permanent DOI.

## Footnotes

1 The slight change in ZFS parameters for diF-TES-ADT in drop-cast films and in solution is likely due to effects of the different molecular environment on the spin density distribution.

## Notes

Sample preparation and characterization, experimental methods, steady-state absorption and emission spectra, temperature-dependent time-resolved PL dynamics, trESR experimental details and modeling, and temperature-dependent MFE experimental details and modeling (PDF)
