# Supplementary material for: Intersystem Crossing Outcompetes Triplet-Pair Separation from 1(TT) below 270 K in Anthradithiophene Films
Source: J Am Chem Soc. 2025 Jul 30;147(32):28638–50. doi: 10.1021/jacs.5c00001 (PMC12356544; doi:10.1021/jacs.5c00001)
Supplement: Supplementary file 1 [file ja5c00001_si_001.pdf]

Supporting Information: Intersystem crossing out-competes triplet-pair separation from ^{1}(TT) below 270 K in anthradithiophene films

Eman M Bu Ali,[†,‡] Arnau Bertran,[¶] Gabriel Moise,[¶] Shuangqing Wang,[§,†] Rachel C Kilbride,[†] John E. Anthony,[‖] Claudia E. Tait,[*,¶] and Jenny Clark[*,†]

†*School of Mathematical and Physical Sciences, The University of Sheffield, Sheffield S3 7RH, UK*

‡*Department of Physics, College of Science, King Faisal University, Al-Hassa, Hofuf 31982, Saudi Arabia*

¶*Department of Chemistry, University of Oxford, Oxford OX1 3QR, United Kingdom*

§*Department of Chemistry and Biochemistry, University of California San Diego, La Jolla, CA 92093, USA*

‖*Department of Chemistry, University of Kentucky, Lexington, KY 40511, USA*

E-mail: claudia.tait@chem.ox.ac.uk; jenny.clark@sheffield.ac.uk

Contents

1 Sample Preparation and Characterization **S-4**

1.1 Preparation of diF-TES-ADT Films S-4

1.1.1 Crystalline film preparation for magnetic field effect and time-resolved photoluminescence measurements S-4

1.1.2 Thin film preparation for steady-state absorption and photoluminescence S-4

1.1.3 Sample preparation for transient ESR measurements S-4

1.2 Morphological Characterization of diF-TES-ADT Films S-5

2 Experimental Methods **S-7**

2.1 Grazing incidence wide-angle X-ray scattering S-7

2.2 Atomic force microscopy S-7

2.3 Ground-state absorption S-7

2.4 Temperature-dependent steady-state PL, time-resolved PL dynamics, and magnetic field effect S-8

2.5 Transient Electron Spin Resonance S-8

2.6 Density Functional Theory calculations S-9

3 Steady-State Absorption and Emission **S-10**

4 Temperature-Dependent Time-Resolved Photoluminescence Dynamics **S-12**

5 Temperature-Dependent MFE measurements **S-13**

6 Transient Electron Spin Resonance **S-16**

6.1 trESR measurements on diF-TES-ADT in frozen solution S-16

6.2 Spectral signatures for photoinduced paramagnetic states for different formation mechanisms S-17

6.3 Partial ordering in diF-TES-ADT films S-18
6.4 Contributions to the trESR spectra S-19
6.5 Modeling of the trESR time evolution S-20

7 Simulation and modeling of MFEs **S-26**

7.1 Kinetic Modeling of Room Temperature MFE S-26
7.2 Kinetic Modeling of Temperature-Dependent MFE S-28
7.3 Fluence-dependent simulation S-34

1 Sample Preparation and Characterization

1.1 Preparation of diF-TES-ADT Films

1.1.1 Crystalline film preparation for magnetic field effect and time-resolved photoluminescence measurements

For MFE and trPL measurements, a drop-cast diF-TES-ADT film was prepared by dissolving a concentration of 15 mg ml^{-1} in toluene to make the solution. A pre-cleaned quartz-coated glass substrate was preheated using a hot plate at 50 °C for a few seconds before drop-casting the solution. A 100 µL volume of the diF-TES-ADT solution was drop cast onto the substrate and allowed to evaporate for $\sim$ 15 minutes on the hot plate to improve the crystallinity of the film (Fig. S1b). The sample was prepared inside a nitrogen-filled glovebox, where all films and solutions preparations took place.

1.1.2 Thin film preparation for steady-state absorption and photoluminescence

Spin-coated thin films were used to perform absorption and emission measurements. The solution was prepared by dissolving a concentration of 15 mg ml^{-1} in anhydrous toluene. 20 µL of the solution was spin-coated onto a pre-cleaned quartz-coated glass substrate at 1200 rpm for 50 seconds. The samples were encapsulated inside nitrogen-filled gloveboxes to reduce photo-degradation. The films were then also stored in a nitrogen-filled glove box to reduce any risk of exposure to oxygen and light.

1.1.3 Sample preparation for transient ESR measurements

Thin film samples for transient ESR experiments were prepared by distributing 5 µL of a 15 mg ml^{-1} solution of diF-TES-ADT in anhydrous toluene onto 2.1 mm x 17 mm pre-cleaned quartz substrates. The solvent was allowed to evaporate at ambient pressure and temperature within a nitrogen-filled glovebox. Two quartz substrates with drop-cast film

were then placed back-to-back within a 2.9 mm OD, 2.5 mm ID quartz EPR tube, back-filled with He to 500 mbar and flame-sealed.

Reference transient ESR measurements were also performed on frozen solution samples. A 250 µM solution of diF-TES-ADT was prepared in anhydrous toluene, 70 µL were transferred to a 3.8 mm OD, 3.0 mm ID quartz EPR tube and the sample was flame-sealed after several freeze-pump-thaw cycles.

1.2 Morphological Characterization of diF-TES-ADT Films

Figure S1 shows grazing incidence wide-angle X-ray scattering GIWAXS (left), polarized microscope (middle), and atomic force microscopy AFM (right) images of (a) spin-coated and (b) drop-cast diF-TES-ADT films. Spin-coated thin film displays micron-scale crystalline texture (Fig. S1a). The distinct grains apparent in the AFM are significantly smaller in the spin-coated film and show stronger preferential orientation with respect to the substrate, as evidenced by the appearance of Bragg spots (in comparison to the broader arc/ ring like features seen in the drop-cast sample). On the other hand, diF-TES-ADT drop-cast film crystallizes into large domains that are dispersed across the substrate (Fig. S1b). We see that these crystalline domains are hundreds of micrometers in size. Several distinct scattering peaks are observed corresponding to the diffraction from highly crystalline domains within the diF-TES-ADT film. The structure of these crystalline regions is consistent with the previously reported brickwork packing with a predominantly edge-on lamellar motif as confirmed by GIWAXS (Figure S2).[S1]

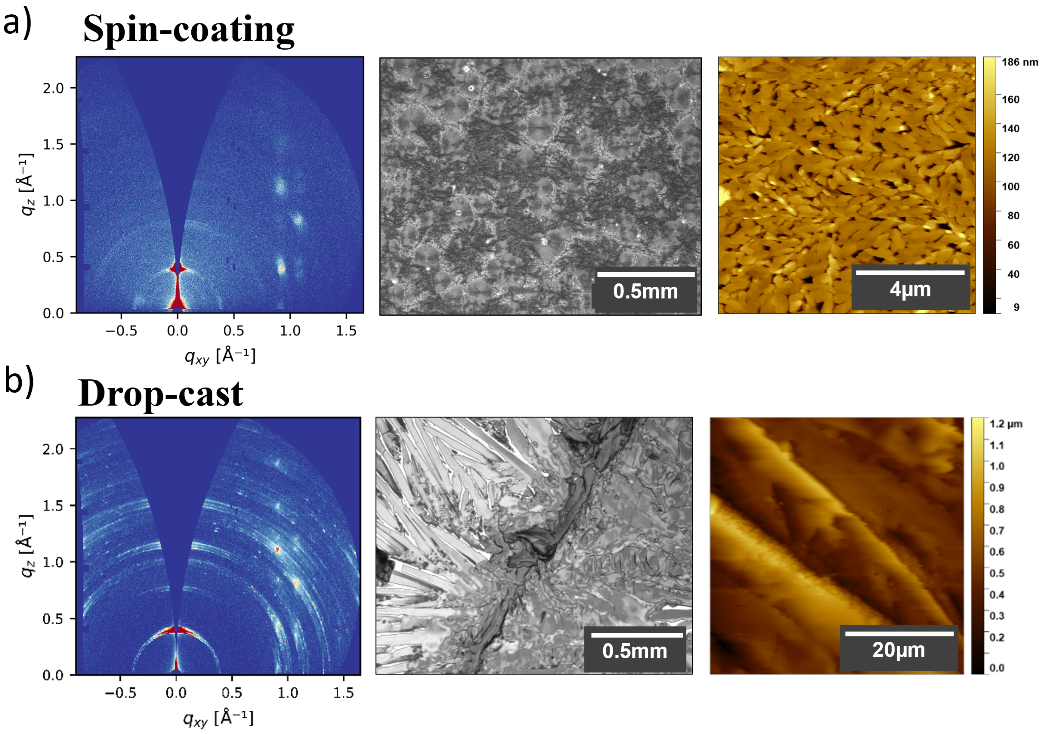

Figure S1: GIWAXS pattern (left), polarized microscope images (middle), and AFM scan (right) of (a) spin-coated and (b) drop-cast diF-TES-ADT films.

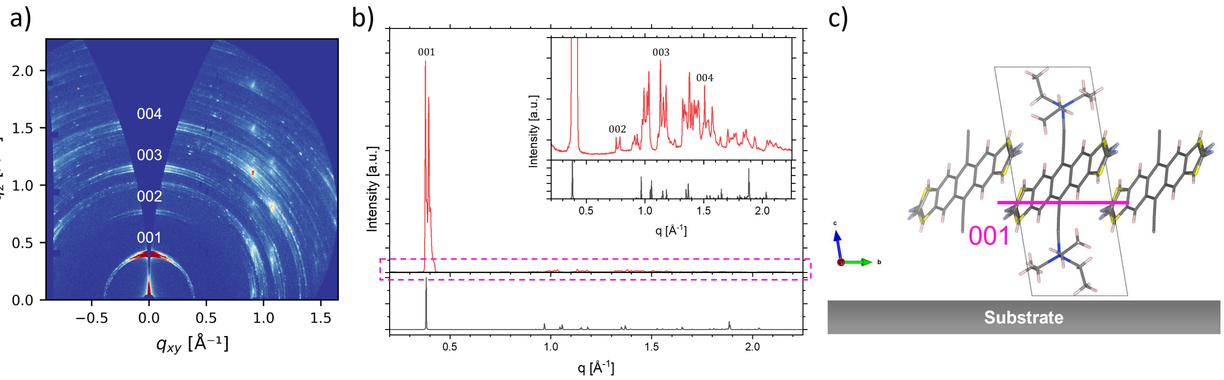

Figure S2: (a) 2D GIWAXS pattern of a drop-cast diF-TES-ADT film (repeated from Figure 1b) with the 001 and higher order reflections labeled. (b) Corresponding 1D GIWAXS intensity profile and simulated powder XRD of the reported crystal structure,[S1] simulated using VESTA software.[S2] The inset shows the region highlighted by the pink dashed rectangle. 1D GIWAXS intensity profiles were generated by azimuthally integrating the 2D pattern across the full q and azimuthal ranges. Here $q = 4\pi \sin\theta/\lambda$, where 2θ is the angle between the incident and scattered X-ray of wavelength λ and the azimuthal angle is the angle normal to the beam incidence at the detector. The out-of-plane scattering is dominated by a feature corresponding to the (001) plane, indicating a predominantly lamellar textured film (edge-on motif) with (001) planes aligned parallel to the substrate, as illustrated in (c).

2 Experimental Methods

2.1 Grazing incidence wide-angle X-ray scattering

GIWAXS measurements were performed using a Xeuss 2.0 laboratory beamline (Xenocs) equipped with a liquid gallium MetalJet source (Excillum), producing X-rays with an energy of 9.243 keV (λ= 1.34 Å) . A collimated X-ray beam was directed at sample surfaces inclined at a grazing angle of 0.15° and scattered X-rays were detected by a Pilatus3R 1M 2D X-ray detector (Dectris) positioned ∼307 mm from the sample center. The sample-to-detector distance was calibrated using a silver behenate standard in transmission geometry. During measurement, the entire flight path including collimation tubes and sample chamber were held under vacuum to minimize background air scatter. GIWAXS data were corrected, reshaped, and reduced using code based on pyFAI and pygix python libraries.[S3]

2.2 Atomic force microscopy

AFM measurements were performed using a Dimension 3100 (Veeco) microscope, equipped with a Nanoscope 3 A feedback controller. Scout 350 RAl (NuNano) cantilevers were used with a resonant frequency of 350 kHz and spring constant of 42 N/m. The data was processed using Gwyddion software (Version 2.60).[S4]

2.3 Ground-state absorption

Ground state absorption measurement of diF-TES-ADT thin film was conducted at room temperature using a UV-visible spectrophotometer (Cary60, Agilent).

2.4 Temperature-dependent steady-state PL, time-resolved PL dynamics, and magnetic field effect

A home-built setup was used for temperature-dependent steady-state PL, trPL, and MFE measurements. The sample was mounted in a closed-cycle He cryostat (Magneto-optic module, Montana Instruments), which controls both magnetic field intensity and temperature applied to the sample. This cryostat offers access to a 0.7 T bi-polar magnetic field. The sample is thus placed on a cold flange between the poles using double-sided tape, with a radiation shield, a vacuum housing with integrated poles, and an electromagnet installed to initiate cooling. Once cooled, bipolar field strength can be controlled. It's worth mentioning that the temperature shown on the cryostat reflects the temperature at the cold flange, where cooling is directly applied. The sample itself might not be exactly at this temperature, particularly at very low temperatures. The difference results from imperfect thermal contact between the sample and the cold flange. A frequency-doubled output Q-switched Nd: YVO4 laser (Picolo-AOT, Innolas) was used to excite the sample. This laser generated 5 kHz pulses with a temporal width of 500 ps and a wavelength of 532 nm. Spectra were recorded using a time-gated intensified charge-coupled device (iCCD; iStar DH334T-18U-73, Andor). A 532 nm notch filter and two 550-colored glass long-pass filters (OG550, Schott) were placed in front of the detector for the PL measurements.

2.5 Transient Electron Spin Resonance

Transient ESR experiments were performed on a Bruker Elexsys E680 X-band spectrometer equipped with a Bruker ER- 4118X-MD5-W1 dielectric resonator with an optical window. Measurements at a series of temperatures in the range from 20 K to 250 K were performed using liquid helium or liquid nitrogen cooling with an Oxford Instruments CF935 cryostat and temperature-control system. Laser excitation at 532 nm was provided by an EKSPLA NT230 diode pumped Q-switched Nd:YAG laser and optical parameteric oscillator (OPO)

with 5 ns pulses and a 50 Hz repetition rate. After the last laser turning mirror, the light was depolarized using an achromatic depolarizer. A Stanford Research System digital delay generator (DG645) was used for synchronization of the laser and EPR spectrometer. The laser energy incident on the sample was estimated based on laser energy measurements within the resonator and cryostat using a ThorLabs TD2X thermal detector placed at the position of the sample (after calibration outside the resonator). Transient EPR experiments were performed by direct detection with the transient recorder (Video Amplifier III, 1 GHz bandwidth) without lock-in amplification using a microwave power of 2 mW. The laser background signal was removed by 2D baseline-correction determined based on low- and high-field off-resonance transients.

2.6 Density Functional Theory calculations

DFT calculations were performed to predict the orientation of the principal axes of the zero-field interaction with respect to the molecular structure. Geometry optimizations for the ground and triplet state were performed in ORCA (v.5.0.4)[S5] with the BP86 basis set and the def2-SVP basis set, including a dispersion correction,[S6–S9] EPR parameters were calculated for the optimized geometry with the PBE0 basis set and EPR-II basis for H, C and N and the IGLO-III basis set for S.[S10–S13] The calculated spin density distribution and principal axis orientations are depicted in Fig. S11. DFT predicts a positive zero-field splitting D value with the Z principal axis along the out-of-plane direction, in agreement with other polyacene-based molecules.

3 Steady-State Absorption and Emission

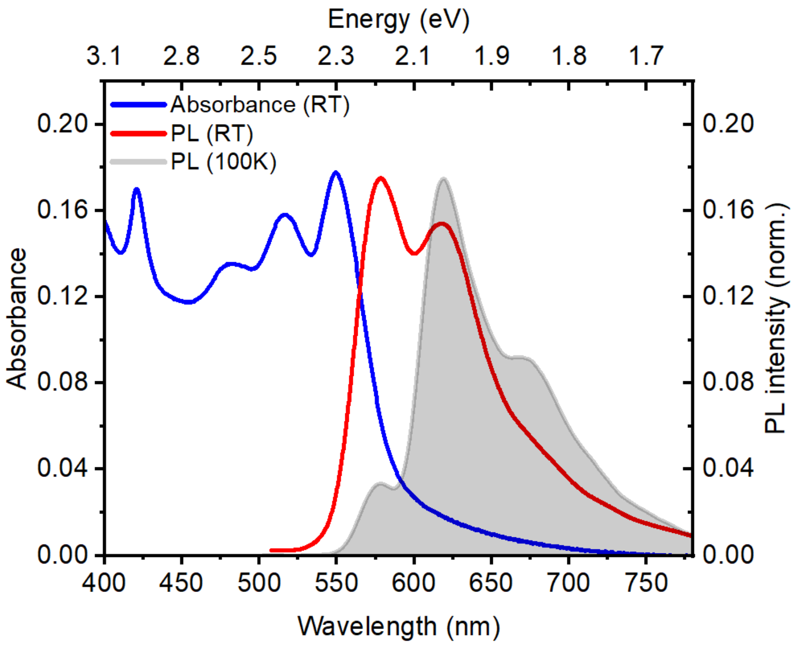

Figure S3: diF-TES-ADT thin film steady-state room temperature absorption spectrum (blue) and photoluminescence (PL) spectra at room temperature (red) and 100 K (gray shaded).

Temperature-dependent steady-state PL of the diF-TES-ADT film excited at 532 nm in Figure S4 exhibits an increase of a strongly coupled triplet pair state ^{1}(TT) emission at low temperatures.[S14] This emission is shifted towards slightly longer wavelengths compared to the original emission peak at RT. The contribution from singlet states decreases as the temperature decreases from room temperature to 100 K.

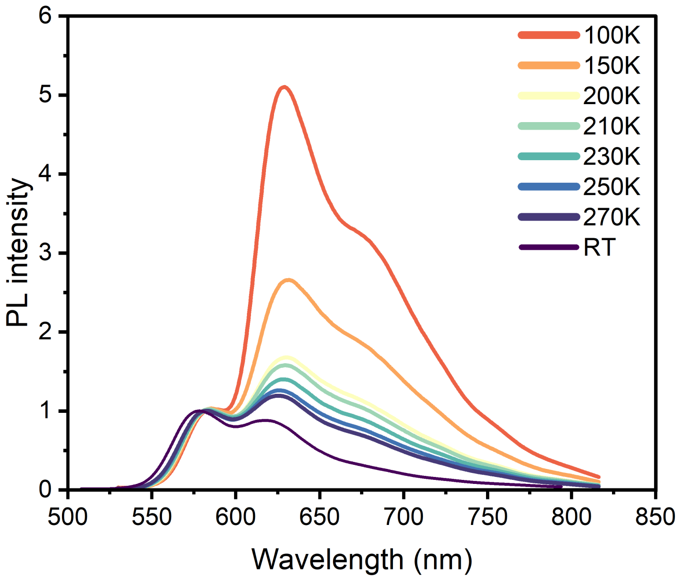

Figure S4: Temperature-dependent steady-state PL of a diF-TES-ADT, excited at 532 nm, exhibits an increase of the ^{1}(TT) emission at low temperatures along with a decrease in the contribution from singlet states.

4 Temperature-Dependent Time-Resolved Photoluminescence Dynamics

TrPL dynamics exhibit minimal temperature dependence within the time constants range from ∼10 to 30 ns, while changing the temperature from 20 K to RT, as illustrated in Figure S5. As previously observed, the PL intensity initially decreases exponentially, characterized by a single time constant of ∼25 ns which is the lifetime of the emissive ^{1}(TT) states. These ^{1}(TT) states, created by singlet fission SF, may either return to the ground state or transform into long-lived triplets.[S15] More intense emission on longer timescales indicates bimolecular triplet-triplet annihilation TTA, as reported in reference.[S14]

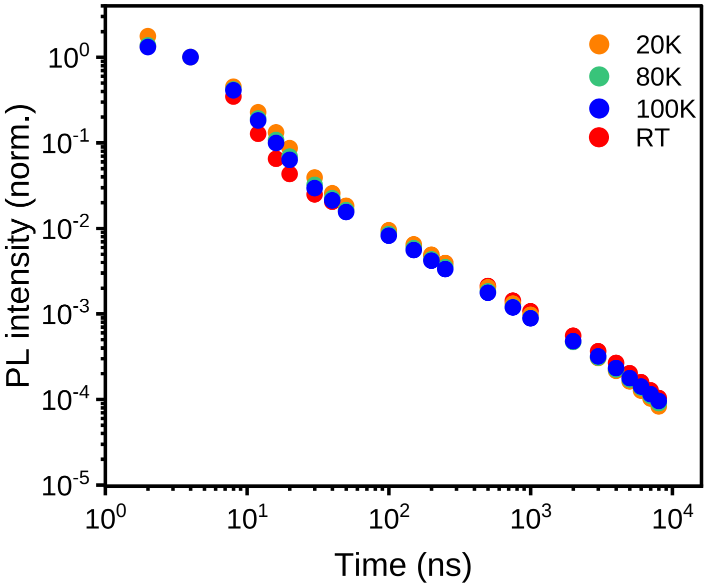

Figure S5: Temperature-dependent time-resolved photoluminescence dynamics of the diF-TES-ADT film, excited at 532 nm. TrPL dynamics exhibit minimal temperature dependence within the time constants range from ∼10 to 30 ns, while changing the temperature from 20 K to RT.

5 Temperature-Dependent MFE measurements

The impact of the magnetic field was evaluated by measuring PL spectra at varied magnetic field strengths ranging from 0 mT to 280 mT, and at various delay times ranging from 5 ns to 1 µs. The PL spectra were recorded while repeatedly changing the magnetic field strength in both upward and downward directions to ensure that the PL spectra obtained in both cases have the exact shape and magnitude. This was done to exclude any potential impacts of sample photo-degradation or laser power fluctuations and to reinforce our confidence in the reliability of the noted effects of the magnetic field. Figure S6 shows some of the recorded PL spectra at RT (Fig. S6a) and 100 K (Fig. S6b), while repeatedly changing the magnetic field strength in both upward (black dots) and downward (lines) directions. At different delay times, the recorded spectra remained consistent in terms of shape and magnitude while increasing and then decreasing the magnetic field strength. The laser power level was monitored using a power meter to evaluate laser stability and record any power fluctuations. This allowed the integration of the whole range of wavelengths and to calculate the MFE using the relevant equation:

$$\frac{\Delta \mathrm{PL}}{\mathrm{PL}}(\mathrm{B}) = \frac{\mathrm{PL}(\mathrm{B}) - \mathrm{PL}(0)}{\mathrm{PL}(0)}$$

Figure S7 illustrates the impact of magnetic field on the PL spectra of the diF-TES-ADT crystal at room temperature and 100 K. By recording PL spectra at different magnetic field strengths, Figure S7(a,b) demonstrates an inverse pattern of PL intensity measured at RT. This variation is observed between early and later delay times, 10-20 ns and 350-500 ns, respectively. In contrast, at 100 K, although there is a variation in the magnetic field strength, we observe no corresponding change in the emission intensity throughout the earliest delay time (Fig. S7c). However, at a later time, 350-500 nm, changing the magnetic field strength caused a noticeable change in the emission intensity (Fig. S7d), which is consistent with the recorded data at room temperature. The resulting MFE at various delay times ranging from

5 ns to 1 µs is shown in Figure S8, representing ΔPL/PL(%) as a function of magnetic field strength at room temperature (Fig. S8a) and 100 K (Fig. S8b). See Figures 3 and 4 in the main text.

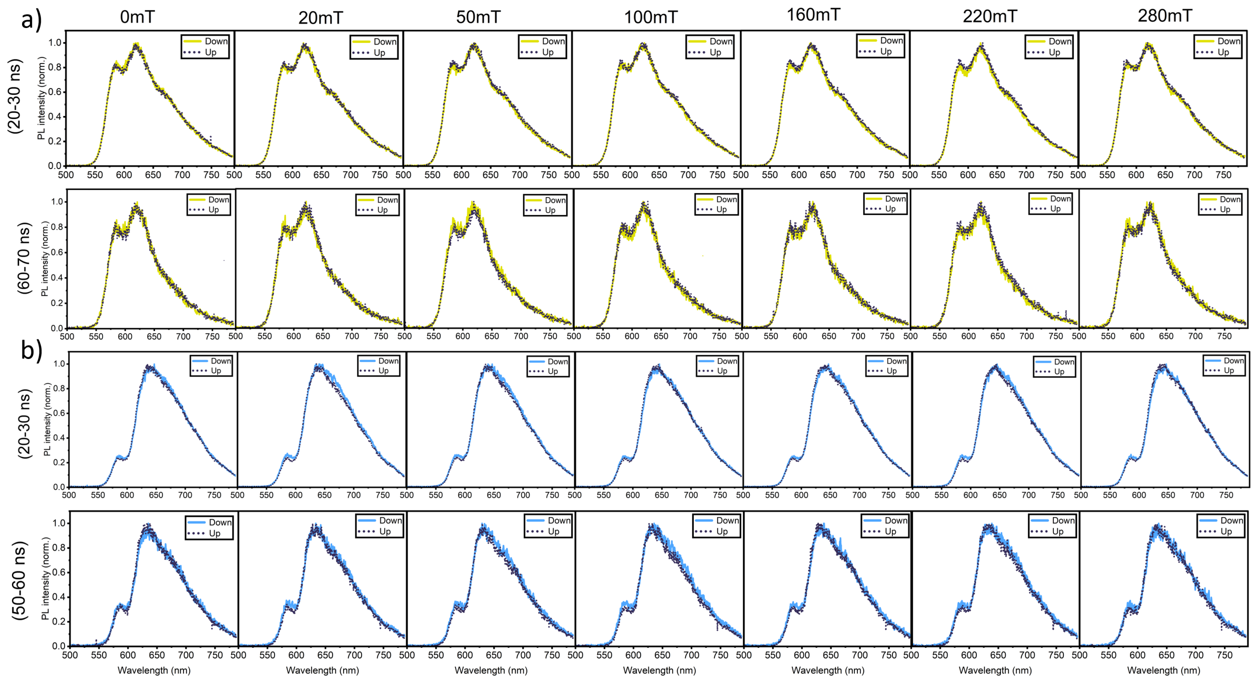

Figure S6: The recorded PL spectra at (a) Room temperature and (b) 100 K while repeatedly changing the magnetic field strength in both upward (black dots) and downward (lines) directions, showing the exact shape and magnitude of the PL spectra in both cases.

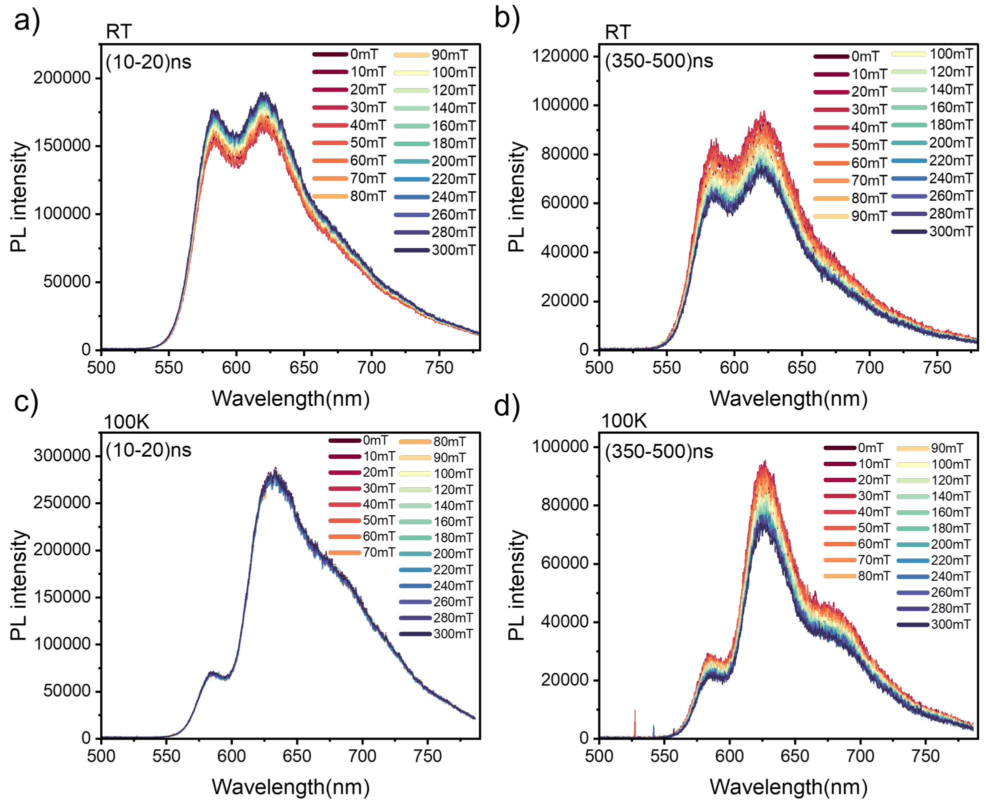

Figure S7: Temperature-dependent Photoluminescence spectra of the diF-TES-ADT drop-cast film as a function of magnetic field strengths measured at (a,b) room temperature, and (c,d) 100 K, at 10-20 ns and 350-500 ns, respectively.

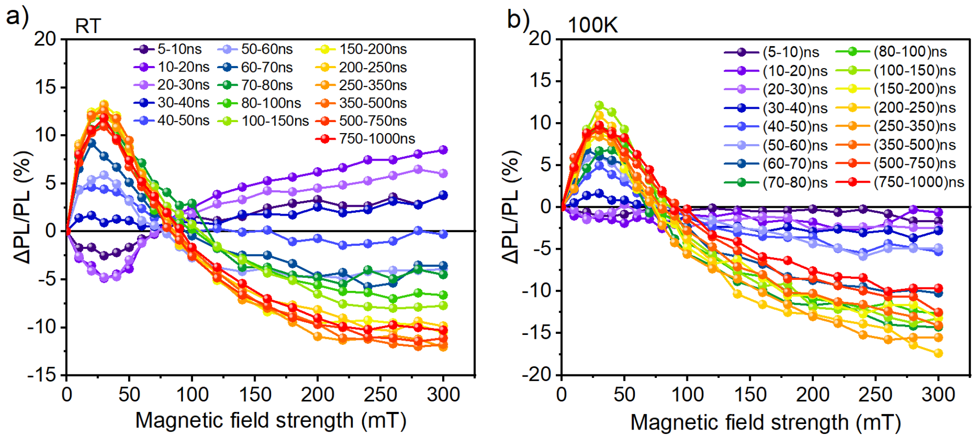

Figure S8: MFE of the diF-TES-ADT drop-cast film measured at 532nm across different delay times, from 5 ns to 1 μs, exhibiting ΔPL/PL(%) as a function of magnetic field strength at (a) Room temperature, and (b) 100 K.

6 Transient Electron Spin Resonance

6.1 trESR measurements on diF-TES-ADT in frozen solution

In order to clearly identify and assign features in ESR spectra attributable to singlet fission or triplet-triplet annihilation, reference measurements were performed on dilute frozen solutions of diF-TES-ADT in toluene, where these processes do not occur. The corresponding trESR spectra are shown in Fig. S9 and show the presence of an intersystem crossing (ISC)-polarized triplet excited state at all temperatures probed. The triplet state ESR signal decays over time while maintaining the initial spin polarization pattern. These results are compared to the results obtained for drop-cast diF-TES-ADT films in Fig. 6 of the main text.

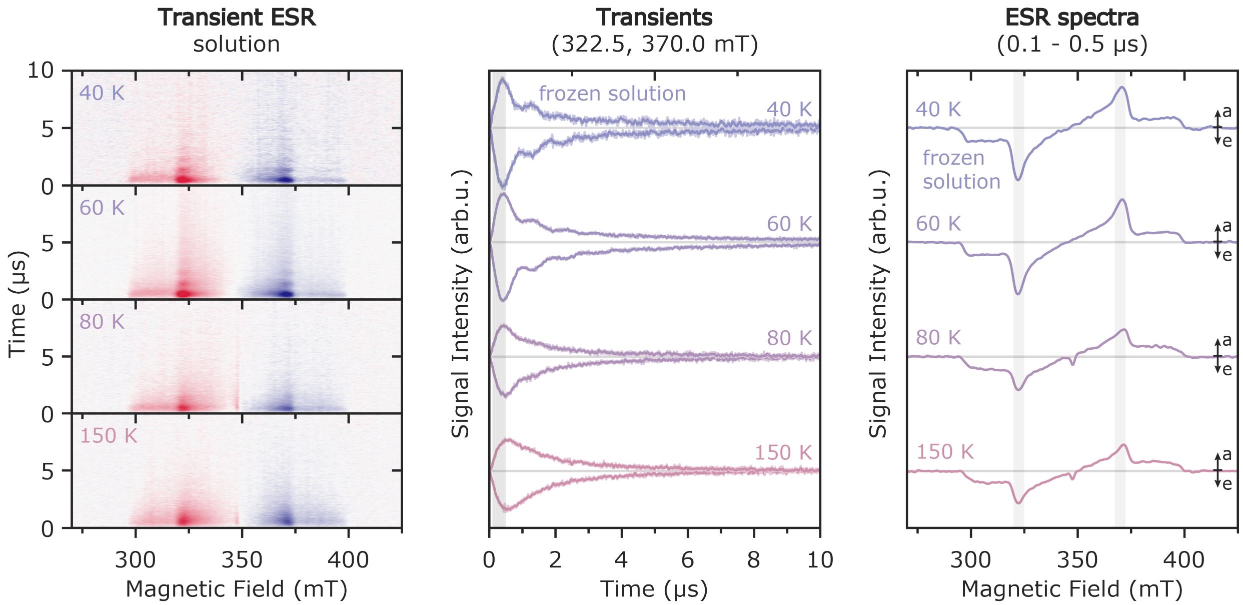

Figure S9: Transient ESR measurements on diF-TES-ADT in frozen solution at a series of temperatures. Time-dependent evolution of the ESR spectra as a function of time after laser excitation (*left*, red = emissive, blue = absorptive), transients extracted at the field positions corresponding to the X canonical field positions (322.5 mT and 370.0 mT, *center*) and spectra extracted at early times after laser excitation (0.1 - 0.5 μs, *right*).

6.2 Spectral signatures for photoinduced paramagnetic states for different formation mechanisms

The spectral signature of the triplet state in diF-TES-ADT resulting from ISC is compared to the expected signatures for triplet and quintet states resulting from singlet fission in Fig. S10. The differences in spin polarization pattern and position of the main spectral features between the ISC and singlet-fission born triplet and quintet states allow unequivocal interpretation of the experimental results in terms of ISC-born triplet states.

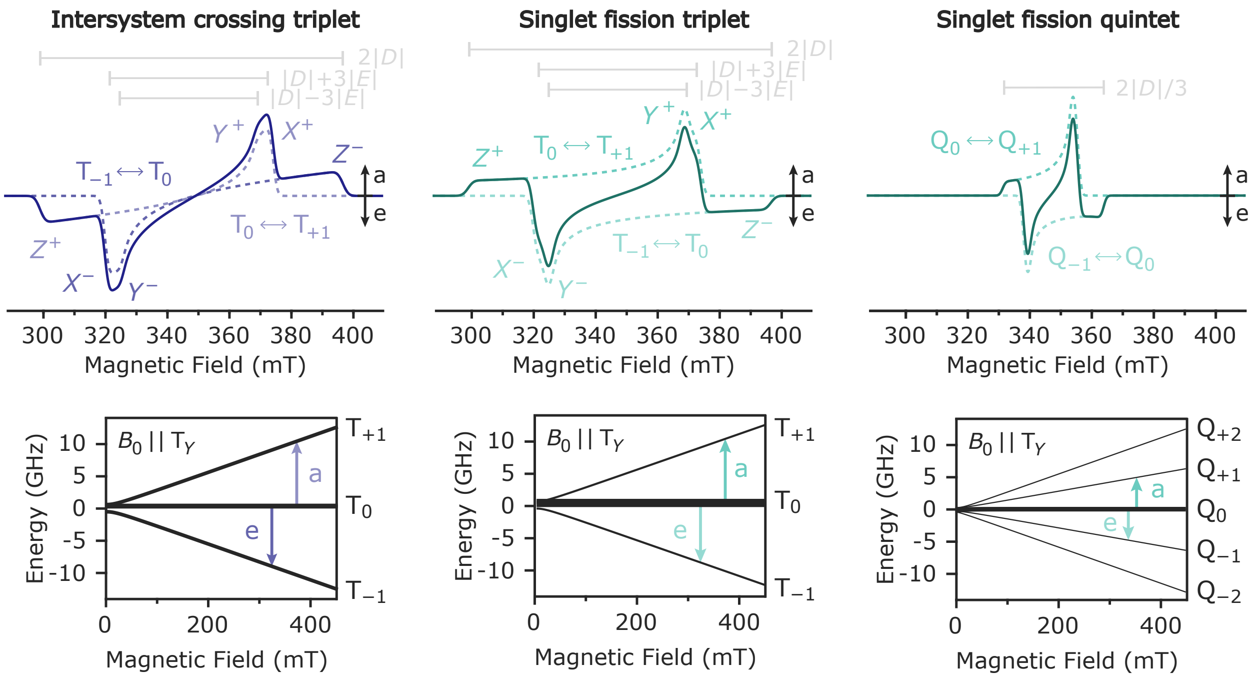

Figure S10: Calculated spectral signatures expected for triplet and quintet states in diF-TES-ADT resulting from different mechanisms: spin-selective population of zero-field populations by intersystem crossing (ISC) and selective population of the $m_S = 0$ sublevel by singlet fission. The contributions of the different transitions are shown as dashed lines and the overall spectrum resulting from their sum is shown as a solid line. The energy level diagrams with line thickness representing relative sublevel populations are shown for a magnetic field aligned with the Y-axis of the zero-field interaction.

6.3 Partial ordering in diF-TES-ADT films

Transient ESR measurements performed on freshly prepared drop-cast diF-TES-ADT films at 20 K show evidence for partial ordering of the diF-TES-ADT molecules in the film as revealed by differences in the ESR spectra recorded with the magnetic field aligned with the substrate plane and with the substrate normal (Fig. S11). Simulation of both the frozen solution and the drop-cast film measurements at the two substrate orientations, combined with information on the orientation of the principal axis frame of the zero-field interaction from DFT, indicates a preferential orientation of diF-TES-ADT molecules with the axis of maximum dipolar coupling (Z), the out-of-plane axis, parallel to the substrate plane and the zero-field Y axis, lying along the TES sidechains, aligned with the substrate normal.

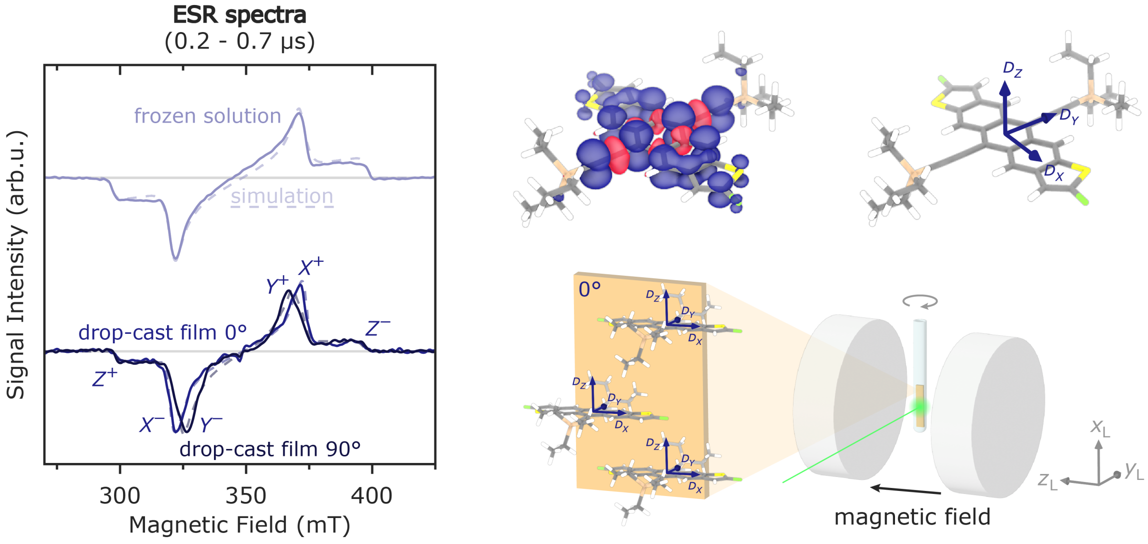

Figure S11: Transient EPR spectra for diF-TES-ADT in frozen solution and in a drop-cast film recorded at 20 K and extracted at early times after photoexcitation(0.2 - 0.7 μs). The spectra are displayed for two different orientations of the film in the spectrometer, 0° corresponds to a magnetic field parallel to the substrate and 90° to the magnetic field along the substrate normal (see drawing on the bottom right). The spin density distribution predicted by DFT for the photoexcited triplet state of diF-TES-ADT and the orientation of the principal axes of the zero-field interaction within the molecular structure are displayed on the top right. The experimental spectra are compared to simulations shown as dashed lines, which for the drop-cast films take a probability distribution of molecular orientations in the film into account.

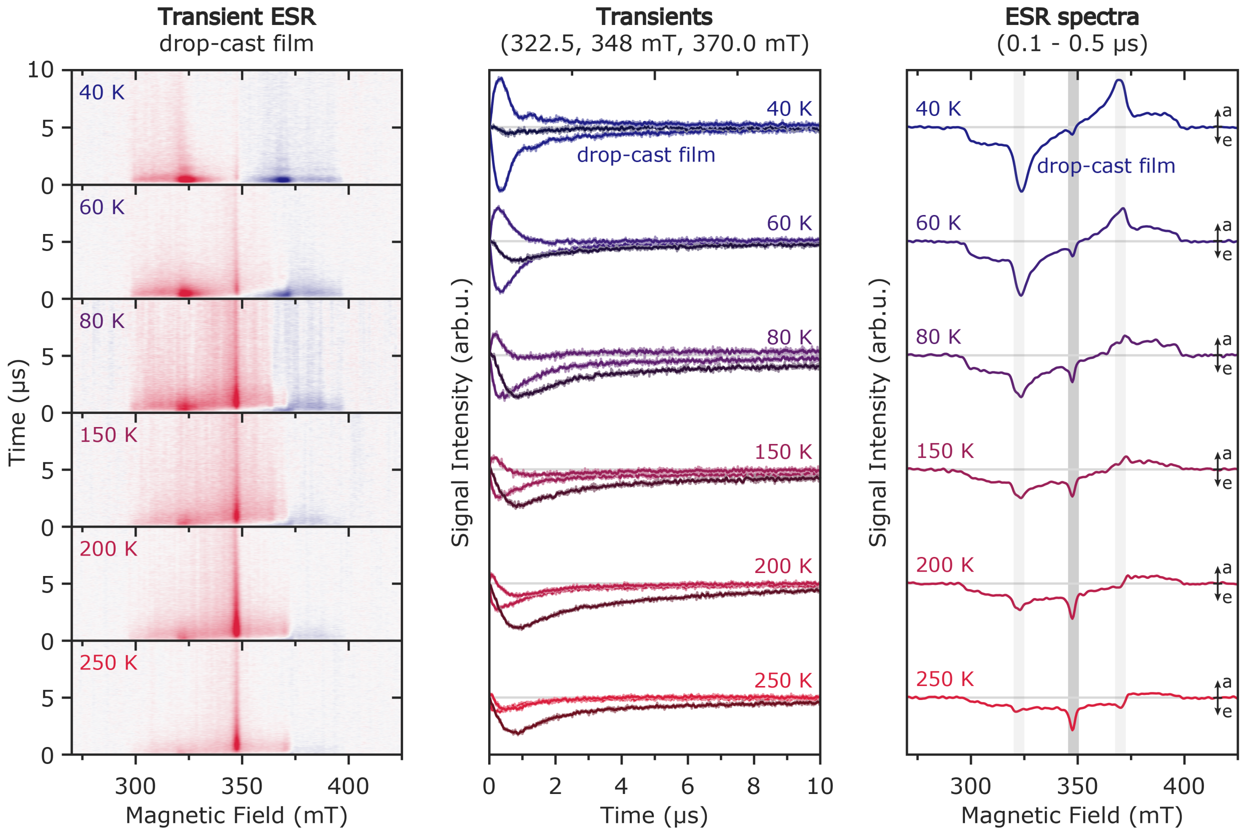

Figure S12: Transient ESR measurements on diF-TES-ADT drop-cast films at a series of temperatures. Time-dependent evolution of the ESR spectra as a function of time after laser excitation (*left*, red = emissive, blue = absorptive), transients extracted at the field positions corresponding to the X canonical field positions (322.5 mT and 370.0 mT) and to the field position of the narrow emissive signal contribution (348 mT, *center*) and spectra extracted at early times after laser excitation (0.1 - 0.5 µs, *right*).

The orientational dependence of the film ESR spectra is in agreement with the preferential edge-on orientation of the diF-TES-ADT molecules determined by GIWAXS, confirming a common morphology between films used for the optical and ESR measurements.

6.4 Contributions to the trESR spectra

The trESR spectra exhibit a sharp emissive signal at $g \approx 2.004$ (347.6 mT), in addition to the broad signal of the photoexcited triplet state. This signal has an approximately Lorentzian lineshape with a FWHM of ca. 1.7 mT and its contribution is more pronounced in the films and increases in intensity for higher temperatures. The rise time of this signal contribution

is slower compared to that of the main triplet ESR signal (see Fig. S12). Potential origins for this signal contribution are a spin-correlated radical pair formed by charge transfer, highly mobile triplets with averaged out dipolar interaction[S16,S17] or a polarized radical state formed following photodegradation. The absence of a signal at the corresponding field position by continuous-wave ESR rules out the thrid option. Attempts at clearly discriminating between a radical and a triplet state origin through pulse or transient nutations were unfortunately not successful.

6.5 Modeling of the trESR time evolution

The time evolution of the spin-polarized ESR signal was simulated to confirm assignment of the observed change in spin polarization pattern to triplet-triplet annihilation and extract the characteristic kinetic parameters describing this process. The kinetic model used for the simulation was adapted from reference[S18] and is shown schematically in Fig. S13. The evolution of the triplet state sublevel populations, N_{+1}, N_0 and N_{-1}, was determined from the solution of the following set of differential equations taking into account spin relaxation, spin-selective unimolecular triplet decay kinetics and bimolecular triplet-triplet annihilation:

$$\begin{aligned}
\frac{\mathrm{d}N_{+1}}{\mathrm{d}t} &= -\,w_{0\leftrightarrow+}N_{+1} \;-w_{-\leftrightarrow+}N_{+1} \;+w_{0\leftrightarrow+}p_{0\leftrightarrow+}N_0 \;+w_{-\leftrightarrow+}p_{-\leftrightarrow+}N_{-1} \\
&\quad -k_{+1}N_{+1} \;-k_{\mathrm{TTA},+1}N_{+1}N_{-1} \\
\frac{\mathrm{d}N_0}{\mathrm{d}t} &= -\,w_{0\leftrightarrow+}p_{0\leftrightarrow+}N_0 \;-w_{-\leftrightarrow0}N_0 + w_{0\leftrightarrow+}N_{+1} \;+w_{-\leftrightarrow0}p_{-\leftrightarrow0}N_{-1} \\
&\quad -k_0N_0 \;-k_{\mathrm{TTA},0}N_0N_0 \\
\frac{\mathrm{d}N_{-1}}{\mathrm{d}t} &= -\,w_{-1\leftrightarrow0}p_{-\leftrightarrow0}N_{-1} \;-w_{-\leftrightarrow+}p_{-\leftrightarrow+}N_{-1} \;+w_{-\leftrightarrow0}N_0 \;+w_{-\leftrightarrow+}N_{+1} \\
&\quad -k_{-1}N_{-1} \;-k_{\mathrm{TTA},-1}N_{+1}N_{-1}
\end{aligned}$$

$w_{i\leftrightarrow j}$ are the relaxation rates for the different level pairs and the factors $p_{i\leftrightarrow j}$

$$p_{i\leftrightarrow j} = \exp\left(-\left(E_j - E_i\right)/k_{\mathrm{B}}T\right)$$

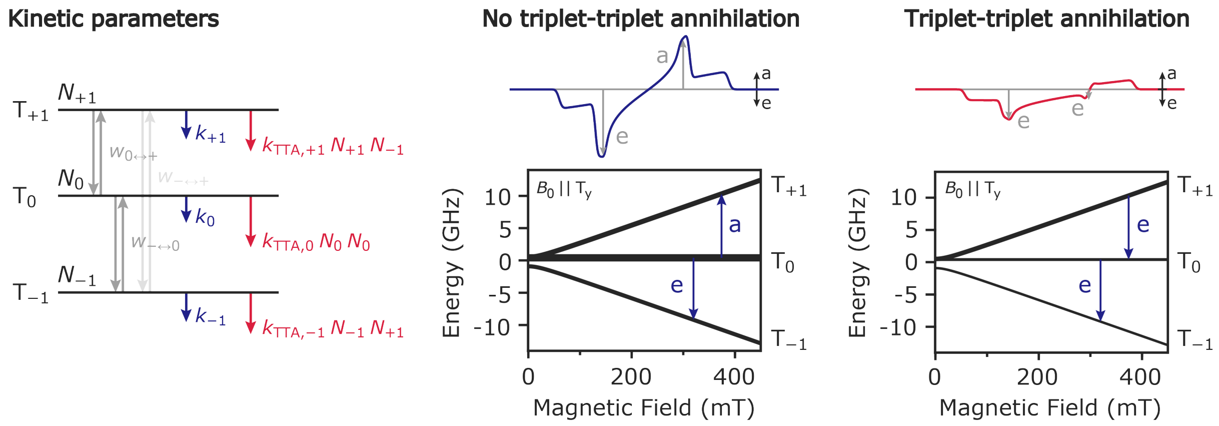

Figure S13: Schematic drawing of the kinetic model used for the trESR simulations (*left*) and illustration of the effect of triplet-triplet annihilation on the triplet sublevel populations and therefore the spin polarization of the ESR spectrum (*right*). The energy level diagrams are shown for a magnetic field aligned with the Y-axis of the zero-field interaction, the orientation most sensitive to the effects of triplet-triplet annihilation for this system.

ensure return to the Boltzmann equilibrium populations.

k_{+1}, k_0 and k_{-1} are the unimolecular triplet decay rates for each high-field triplet sublevel, which are determined from the selective decay rates k_X, k_Y and k_Z in the molecular (zero-field) frame for each molecular orientation as

$$k_i = \sum_l c_i^2 k_l \qquad i = -1, 0, +1 \qquad l = X, Y, Z$$

$k_{\text{TTA},+1}$, $k_{\text{TTA},0}$ and $k_{\text{TTA},-1}$ are the decay rates for the triplet-triplet annihilation process corresponding to each high-field triplet sublevel.

This kinetic model was implemented in a modified version of the EasySpin `resfields` function,[S19,S20] which calculates the triplet sublevel populations for each time point and each orientation in the powder average by solution of the differential equations using Matlab's built-in ODE solver and constructs the ESR spectrum at different times after photoexcitation. The finite rise time of the signal was modeled by multiplication of the calculated transients with a response function determined by the resonator bandwidth for a given resonator Q-value.

In order to reduce the number of variable fitting parameters, and therefore minimize the

risk of overfitting, we made a series of approximations. The relaxation rates for the level pairs corresponding to a $\Delta m_S = \pm 1$ transition were set to be equal ($w_{-\leftrightarrow 0} = w_{0\leftrightarrow +}$) and the relaxation rate for level pairs with $\Delta m_S = \pm 2$ was set to be negligible. The unimolecular decay rates k_X, k_Y and k_Z were assumed to be the same for diF-TES-ADT in frozen solution and in drop-cast films and independent of temperature.

The unimolecular decay rates and the temperature-dependent relaxation rates were determined from a global fit of the frozen solution trESR spectra and transients recorded at 40 K, 60 K, 80 K and 150 K. The simulation parameters are reported in table S1. An excellent agreement with the experimental results could be obtained as shown in Fig. S14. The presence of Torrey oscillations in trESR datasets recorded at low temperatures indicates that the measurement conditions, with the microwave power necessary to acquire data with sufficient signal-to-noise ratio, correspond to the underdamped regime.[S21] As the relaxation times decrease for higher temperature, a transition to the overdamped regime is observed. In both regimes, the decay of the signal is not purely determined by T_1, with T_2 significantly influencing the decay in the underdamped regime and the decay constant only approaching T_1 when $\nu_1^2 T_1 T_2 \ll 1$.[S21] Therefore relaxation rate constants determined from the fit cannot be interpreted in terms of specific relaxation times. However, the purpose of modeling the frozen solution trESR data was to determine the rate constants of all processes except triplet-triplet annihilation, so that these could then be kept fixed for the simulation of the data recorded for the drop-cast films to better isolate the contribution of triplet-triplet annihilation.

For the simulation of the trESR data recorded for diF-TES-ADT in drop-cast films, the bimolecular decay rates for the T_{-1} and T_{+1} sublevels were kept equal ($k_{\mathrm{TTA},-1} = k_{\mathrm{TTA},+1}$) and the ratio $k_{\mathrm{TTA},+1/-1}/k_{\mathrm{TTA},0}$ was kept fixed for all temperatures and laser fluences. The ratio $k_{\mathrm{TTA},+1/-1}/k_{\mathrm{TTA},0}$ and the magnitude of TTA decay were determined from a global fit of the trESR spectra and transients recorded at different temperatures and different laser fluences. The resulting simulations are shown in Fig. S14 and the corresponding simulation

parameters are summarized in table S1. The simulations based on this kinetic model including bimolecular triplet-triplet annihilation are clearly able to reproduce the evolution from a symmetric ISC-populated triplet state spectrum at very early times after photoexcitation to the mostly emissive spectrum with a net polarization within the first 1-4 µs. The transition is most clearly visible at the field position corresponding to the high-field Y canonical transition (ca. 370 mT) and results from an inversion of the relative population of the T_{+1} and T_0 levels due to the spin-selectivity of triplet-triplet annihilation (see Fig. S13). Agreement between experiment and simulation is obtained for increased triplet-triplet annihilation at higher temperatures and higher laser fluences.

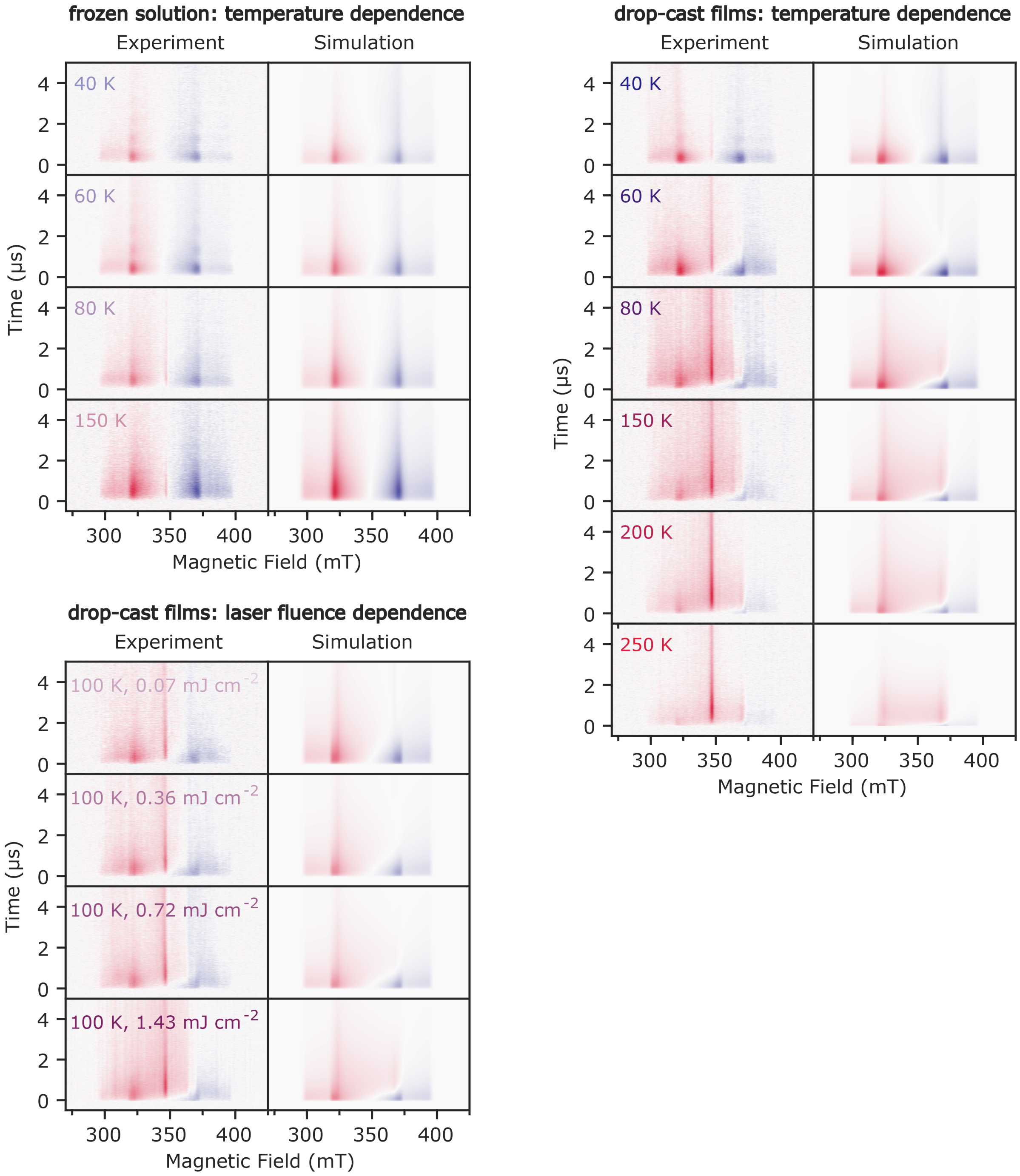

Figure S14: Comparison of the results of the trESR experiments performed as a function of temperature for diF-TES-ADT in frozen solution and as a function of temperature and laser fluence for diF-TES-ADT in drop-cast films with simulations based on a kinetic model taking relaxation, spin-selective unimolecular triplet decay and bimolecular triplet-triplet annihilation into account, performed as described in the text. The simulation parameters are reported in table S1.

Table S1: Parameters for the simulation of ESR spectra and their time dependence.

Spin system parameters

	**frozen solution**	**drop-cast film**
g	2.0059 ± 0.0005	2.0059 ± 0.0005
D	1420 ± 5 MHz	1370 ± 5 MHz
E	-32 ± 2 MHz	-50 ± 5 MHz
D strain	[100 120] MHz	[79 20] MHz
H strain	50 MHz	50 MHz

Spin-selective triplet population and decay

$p_X : p_Y : p_Z$	0.45:0.36:0.19 (±0.02)
$k_X : k_Y : k_Z$	0.32:0.26:0.41 (±0.02) μs^{-1}

Relaxation

	40 K	60 K	80 K	100 K	150 K	200 K	250 K
$w_{-\leftrightarrow 0} = w_{0\leftrightarrow +}$ (μs^{-1})	0.92	0.60	0.33	0.22	0.22	0.50	0.65
$w_{-\leftrightarrow +} = 10^{-4}$ μs^{-1}	$\delta w = 0.05$ μs^{-1}						

Triplet-triplet annihilation*

Temperature	40 K	60 K	80 K	150 K	200 K	250 K
$k_{\mathrm{TTA},+1} = k_{\mathrm{TTA},-1}$ (μs^{-1})	0.42	3.0	7.4	11.0	19.2	38.5
$k_{\mathrm{TTA},0}$ (μs^{-1})	0.47	3.4	8.3	12.3	21.6	43.2

$T = 100$ K

Laser fluence (mJ cm^{-2})	0.07	0.36	0.72	1.43
$k_{\mathrm{TTA},+1} = k_{\mathrm{TTA},-1}$ (μs^{-1})	2.7	3.8	5.5	8.8
$k_{\mathrm{TTA},0}$ (μs^{-1})	3.1	4.3	6.2	9.9

* The ratio $k_{\mathrm{TTA},+1/-1} : k_{\mathrm{TTA},0} (= 0.89 : 1)$ is fixed across all temperatures and laser fluences.

7 Simulation and modeling of MFEs

7.1 Kinetic Modeling of Room Temperature MFE

The initial purpose of Merrifield's model was to describe the magnetic field-dependent fluorescence.[S22] Merrifield's kinetic model was recently modified[S14] to simulate the emission dynamics at room temperature, see reference[S14] for details. The rate model, in this modification, explicitly included two separate populations of triplet-pairs, exchange coupled triplet-pairs ^{1}(TT) and weakly interacting triplet-pairs $(\mathrm{T..T})^l$. This modal includes Merrifield's nine states denoted as $(\mathrm{T..T})^l$ where l=1, 2,..., 9, and their degree of overlap with the singlet is determined by the coefficients $|C_S^l|^2$, which are calculated from the spin Hamiltonian.[S23] Hence, the $|C_S^l|^2$ coefficients change with the magnetic field in accordance with the spin Hamiltonian. The molecule orientation was determined from the crystal structure in reference,[S24] and the zero-field splitting parameters, D and E, were taken from ref.[S15] In addition, it includes spin-lattice relaxation and non-radiative triplet decay from $(\mathrm{T..T})^l$ to $2\times\mathrm{T}_1$. This kinetic model is represented as $\mathrm{S}_1 \rightleftharpoons {}^1(\mathrm{TT}) \rightleftharpoons (\mathrm{T..T}) \rightleftharpoons \mathrm{T}_1 + \mathrm{T}_1$ and shown in Figure S15. In the original publication,[S14] this kinetic model and associated rate equations were used to simulate MFE data recorded at two delay times, 20-30 ns and 100-200 ns, using a custom-made Python code. The governing rate equations are as follows:

$$
\begin{aligned}
\frac{d[\mathrm{S}_1]}{dt} &= -\left(k_{\mathrm{sf}} + k_{\mathrm{snr}}\right)[\mathrm{S}_1] + k_{-\mathrm{sf}}[^1(\mathrm{TT})] \\
\frac{d[^1(\mathrm{TT})]}{dt} &= k_{\mathrm{sf}}[\mathrm{S}_1] - \left(k_{-\mathrm{sf}} + k_{\mathrm{hop}}\sum_{l=1}^{9}|C_{\mathrm{S}}^l|^2 + k_{\mathrm{ttnr}}\right)[^1(\mathrm{TT})] + k_{-\mathrm{hop}}\sum_{l=1}^{9}|C_{\mathrm{S}}^l|^2[(\mathrm{T..T})^l] \\
\frac{d[(\mathrm{T..T})^l]}{dt} &= k_{\mathrm{hop}}|C_S^l|^2[^1(\mathrm{TT})] - \left(k_{-\mathrm{hop}}|C_{\mathrm{S}}^l|^2 + k_{\mathrm{hop2}} + k_{\mathrm{tnr}} + k_{\mathrm{relax}}\right)[(\mathrm{T..T})^l] \\
&\quad + \frac{1}{8}k_{\mathrm{relax}}\sum_{j\neq l}[(\mathrm{T..T})^j] + \frac{1}{9}k_{\mathrm{tta}}[\mathrm{T}_1]^2 \\
\frac{d[\mathrm{T}_1]}{dt} &= \left(k_{\mathrm{tnr}} + 2k_{\mathrm{hop2}}\right)\sum_{l=1}^{9}[(\mathrm{T..T})^l] - 2k_{\mathrm{tta}}[\mathrm{T}_1]^2 - k_{\mathrm{tnr}}[\mathrm{T}_1]
\end{aligned}
$$

The square brackets in these equations represent the concentrations of the states, which are expressed in units of cm^{-3}. The definitions of all rate constants are shown in Figure S15.

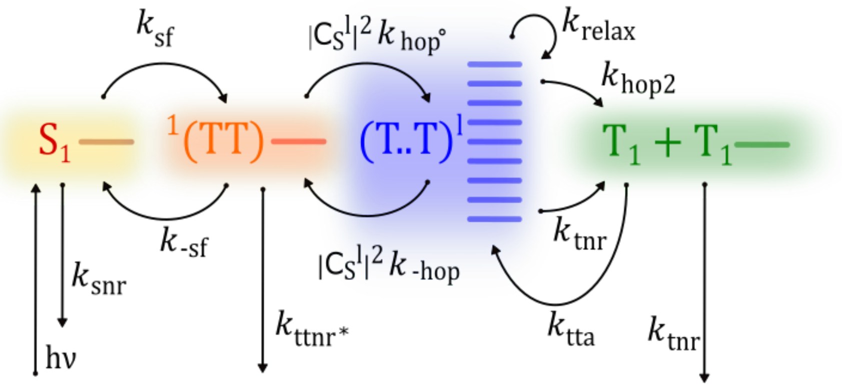

Figure S15: Kinetic scheme of the first modification of Merrifield model which presents SF and TTA behaviors at room temperature.

For our modeling, described below, the initial rate constants k_{SF}, k_{-SF}, k_{hop}, k_{-hop}, k_{relax}, k_{hop2}, k_{tta}, k_{snr}, k_{ssa}, k_{ttnr} and k_{tnr} were taken from the fitting performed in Bossanyi *et al.*,[S14] and used with minimal changes that were well within the constrained values (see supplementary information of reference[S14]).

In that study, the rate constants (k_{sf}, k_{-sf}, k_{snr}) were obtained by fitting the diF-TES-ADT thin film transient absorption data. The remaining seven rate constants were optimized to provide a global fit for a large dataset comprising transient photoluminescence as a function of excitation density and temperature.

Uncertainty analysis of this fitting is shown in the supplementary information of Ref.[S14]

The first step of singlet fission is shorter than the time resolution of the transient PL measurements on which this fitting was based, hence, the parameters k_{sf}, k_{-sf}, and k_{snr} have an insignificant impact on the model's output (but are constrained by the transient absorption data). Furthermore, k_{relax} has no lower bound for temperatures above 100 K, so it only has a significant role at low temperature. Conversely, k_{hop}, k_{-hop}, k_{hop2}, k_{tta}, k_{ttnr}, and k_{tnr} are generally tightly constrained.

Using these rate constants (fitted to and constrained by the transient PL and transient

absorption data), we can *simulate* the room temperature time-resolved magnetic field effect without any fitting. This was our starting point and works reasonably well. Then, with only minor changes to the room temperature rate constants reported below in table S2 for k_{hop}, k_{-hop}, k_{hop2}, and k_{ttnr} (that are well within the bounds described in the uncertainty graphs in[S14]), we more accurately fit the magnetic field effects over all the time ranges measured in the current work.

7.2 Kinetic Modeling of Temperature-Dependent MFE

Here we have further modified the Merrifield kinetic model to include intersystem crossing from ^{1}(TT) and explicitly include temperature-dependence of the triplet-pair separation. Figure S16 presents the kinetic scheme of this newly modified model. This modification involves including an Arrhenius term in the calculation of k_{hop} within the same rate equations used in the kinetic model based on reference.[S14] With fixed parameters from literature,[S14] $k_{hop^*} = k_{hop^\circ} \cdot e^{(\frac{-\Delta E}{k_B T})}$, where k_B is the Boltzmann constant, and ΔE is the activation energy of ΔE=20 meV.[S15] k_{hop° was initially calculated using the value of k_{hop} reported in reference.,[S14] which is equal to 0.0493 ns^{-1}, divided by the factor $e^{(\frac{-\Delta E}{k_B T})}$ at RT.

The second modification of the rate equations involves including intersystem crossing rate constant k_{ISC} to take into account ISC from the ^{1}(TT) state to the T_1 state. The value of this constant was determined by inputting it into the code starting from zero and gradually increasing it until our simulations closely matched the actual data, as illustrated in Figure S17 and S18. Lastly, here we use k_{ttnr^*} to distinguish it from the rate used by reference[S14] who implicitly included k_{ISC} within the non-radiative decay of ^{1}(TT), which they called k_{ttnr}.

The governing modified rate equations used in this model are as follows (where $k_{ttnr^*} = k_{ttnr} - k_{ISC}$ when using k_{ttnr} from reference[S14]):

$$\frac{d[\mathrm{S}_1]}{dt} = -\left(k_{\mathrm{sf}} + k_{\mathrm{snr}}\right)[\mathrm{S}_1] + k_{-\mathrm{sf}}[^1(\mathrm{TT})]$$

$$\frac{d[^1(\mathrm{TT})]}{dt} = k_{\mathrm{sf}}[\mathrm{S}_1] - \left(k_{-\mathrm{sf}} + k_{\mathrm{ISC}} + k_{\mathrm{hop}^*}\sum_{l=1}^{9}|C_{\mathrm{S}}^{l}|^2 + k_{\mathrm{ttnr}^*}\right)[^1(\mathrm{TT})] + k_{-\mathrm{hop}}\sum_{l=1}^{9}|C_{\mathrm{S}}^{l}|^2[(\mathrm{T..T})^l]$$

$$\frac{d[(\mathrm{T..T})^l]}{dt} = k_{\mathrm{hop}^*}|C_S^l|^2[^1(\mathrm{TT})] - \left(k_{-\mathrm{hop}}|C_{\mathrm{S}}^l|^2 + k_{\mathrm{hop2}} + k_{\mathrm{tnr}} + k_{\mathrm{relax}}\right)[(\mathrm{T..T})^l] + \frac{1}{9}k_{\mathrm{tta}}[\mathrm{T}_1]^2 + \frac{1}{8}k_{\mathrm{relax}}\sum_{j\neq l}[(\mathrm{T..T})^j]$$

$$\frac{d[\mathrm{T}_1]}{dt} = (k_{\mathrm{tnr}} + 2k_{\mathrm{hop2}})\sum_{l=1}^{9}[(\mathrm{T..T})^l] + k_{\mathrm{ISC}}[^1(\mathrm{TT})] - 2k_{\mathrm{tta}}[\mathrm{T}_1]^2 - k_{\mathrm{tnr}}[\mathrm{T}_1]$$

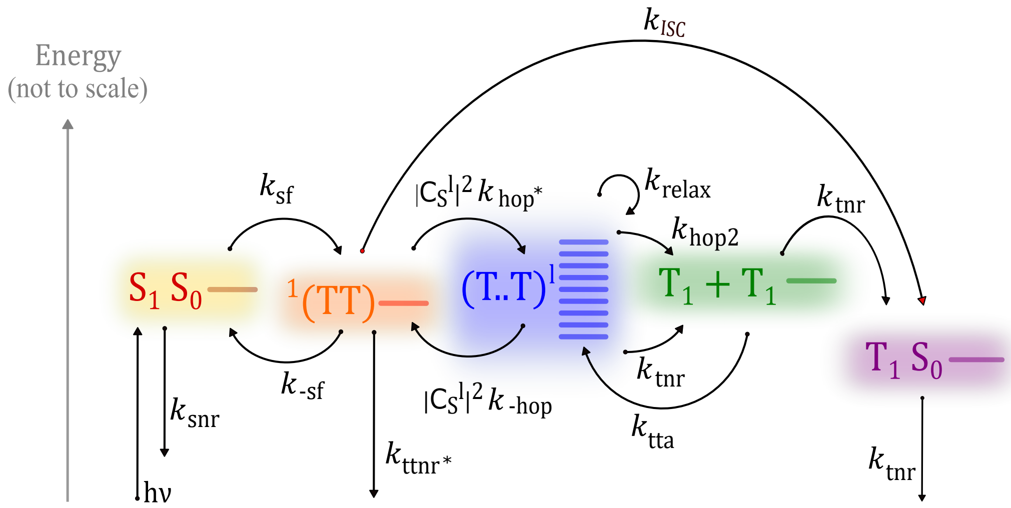

Figure S16: The updated kinetic scheme involving singlet fission at temperatures ranging from 270 K to RT and intersystem crossing at temperatures below 270 K. The rates used in our model are marked in the figure. Here k_{snr}, k_{ttnr*} and k_{tnr} include both radiative and non-radiative decay to the ground-state. The relative energies are NOT to scale (separation between T..T levels is on the order of 1-10μeV, and exchange energy 2J between S_0S_1/S_0T_1 is on the order of 1 eV, while the difference between ^{1}(TT) and T_1+T_1 is ~30 meV).

By applying the updated kinetic model and its associated rate equations, we initiated our simulation with the initial rate constant values from reference[S14] (Table S2). By raising the intersystem crossing rate constant value gradually and observing its impact on the curves across the entire time range, we obtain the results presented in Figure S17 and S18. The

simulation of the MFE at RT (Fig.S17) and 100 K (Fig.S18) demonstrates that when the intersystem crossing rate constant k_{ISC} is 0.17 ns^{-1}, we see similar MFE behavior as in the experimental data. Increasing k_{ISC} further improves the MFE shapes until they reach a saturation point at higher values. The rate constants were adjusted to fine-tune the intensity of the curves to closely match the actual data. The final result of the optimized MFE simulation is shown in Figure S19, which is based on the optimized numbers provided in Table S2.

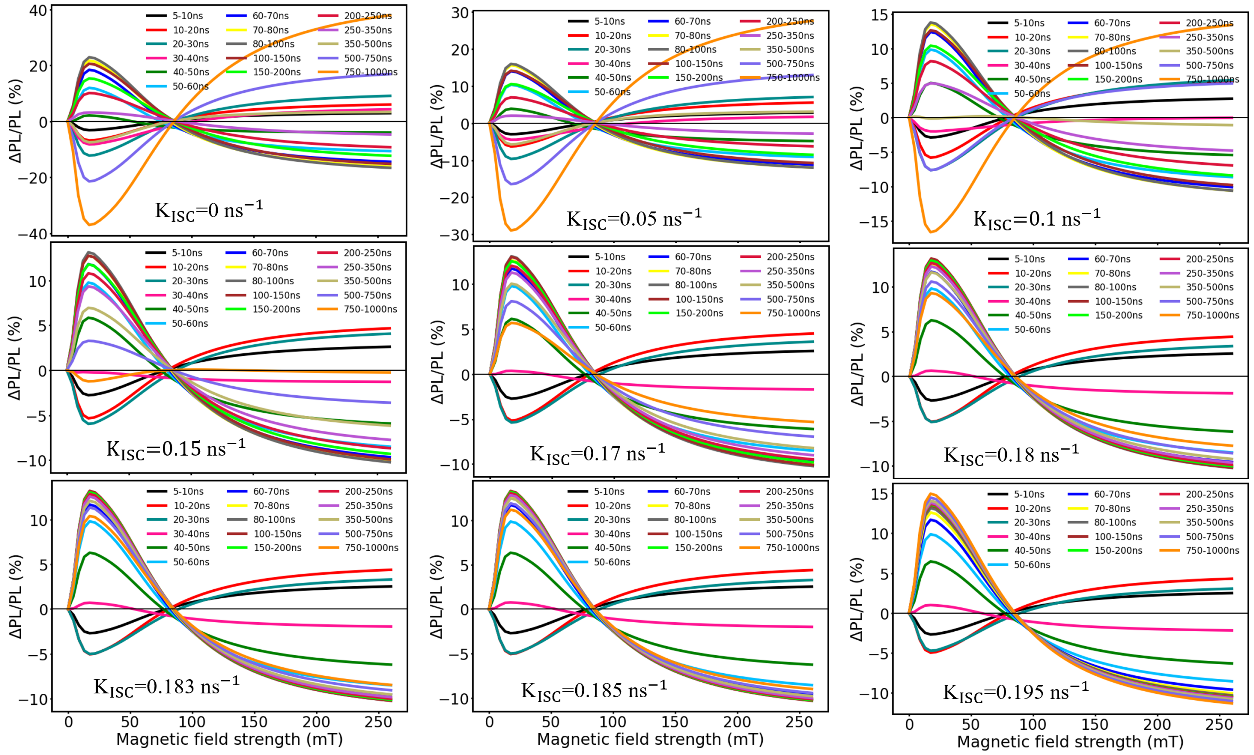

Figure S17: MFE simulation at RT illustrating the impact of a rising intersystem crossing rate constant (k_{ISC}) value on the behavior of the curves across the entire time range.

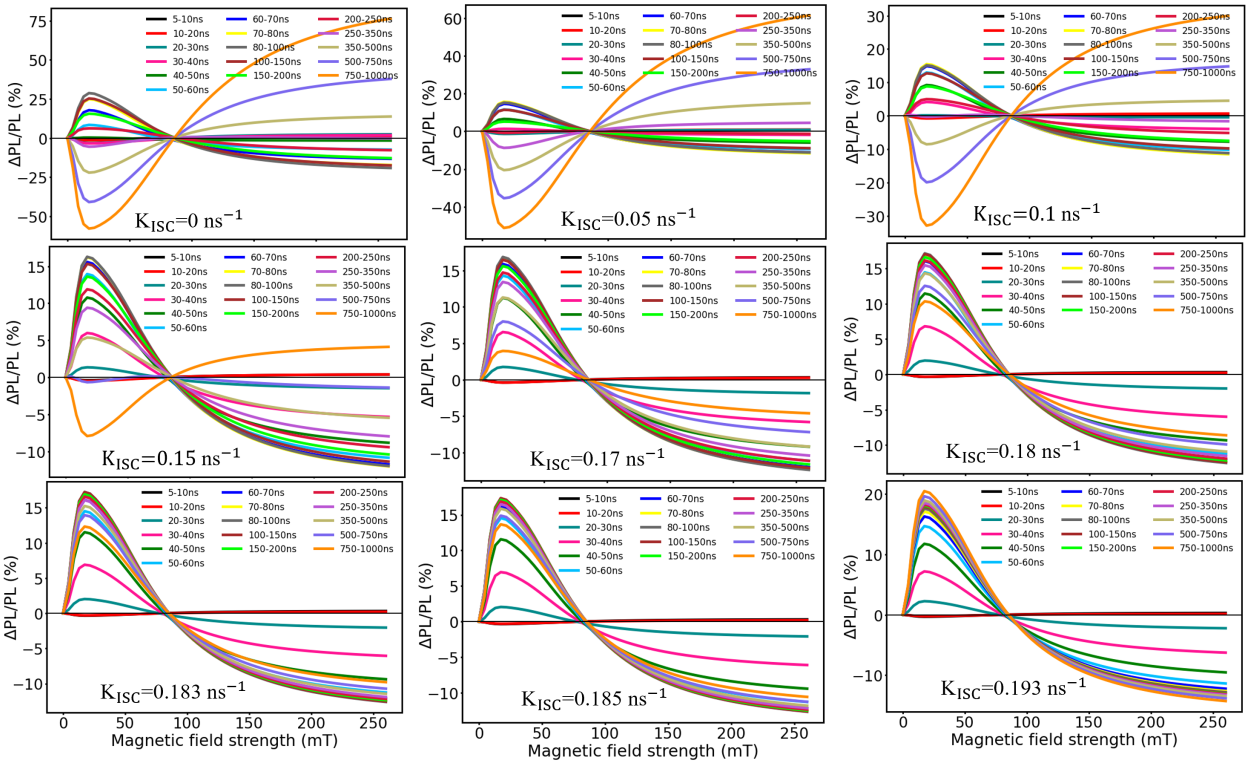

Figure S18: MFE simulation at 100K illustrating the impact of a rising intersystem crossing rate constant (k_{ISC}) value on the behavior of the curves across the entire time range.

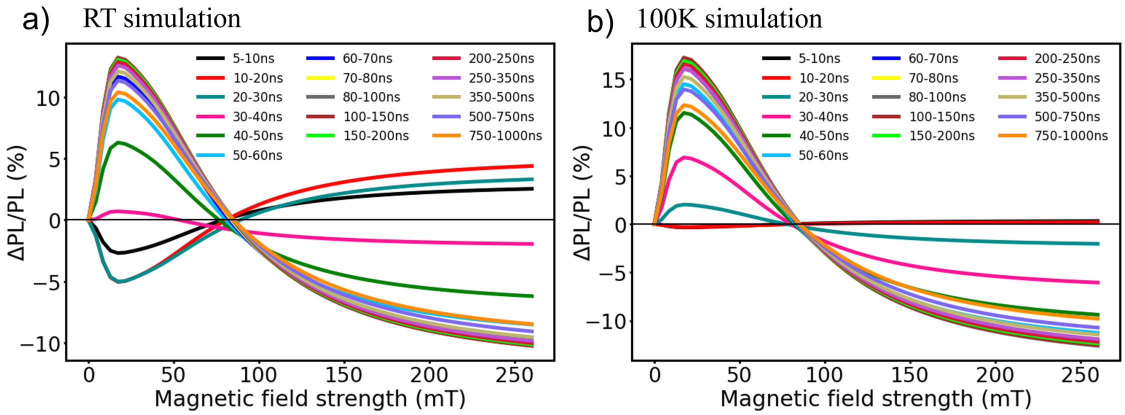

Figure S19: The final result of the MFE simulation at (a) RT and (b) 100 K using the optimized rate constants in Table S2.

Table S2: The initial rate constant values from reference[S14] based on fitting time-, fluence- and temperature-dependent PL of diF-TES-ADT films (left column) and the optimized rate values used in this work to simulate MFE data at RT, 100 K, and power-dependent data. The rate constants are as described in Fig. 9 in the main text. Note that in our rate model, the temperature-dependent k_{hop} rate is replaced by a thermally-activated rate $k_{hop^*}=k_{hop^0}\exp(-E_A/k_BT)$ using $E_A=20\,\mathrm{meV}$ from Ref.[S15]

Rate	Initial values (ns^{-1})	Optimized values (ns^{-1})
k_{gen}	1.8	1.8
k_{sf}	10	10
k_{-sf}	0.083	0.083
k_{hop} /k_{hop^*}	0.0493	0.0493
k_{-hop}	0.0906	0.1106
k_{relax}	0	0
k_{hop2}	0.0644	0.0701
k_{tta}	5.43×10^{-20}	5.43×10^{-20}
k_{snr}	0.083	0.083
k_{ssa}	0	0
k_{ttnr}	0.0948	0.0948
k_{tnr}	1.68×10^{-6}	1.68×10^{-6}
k_{ISC}	-	0.183

Figure S20 displays the derived MFE data and the simulation of diF-TES-ADT drop-cast film at room temperature (S20 a, b), and 100 K (S20 c, d). As presented in this Figure, across all measured delay times, we find that the simulation and the experimental data are in good agreement in terms of shape, intensity, and zero-crossing.

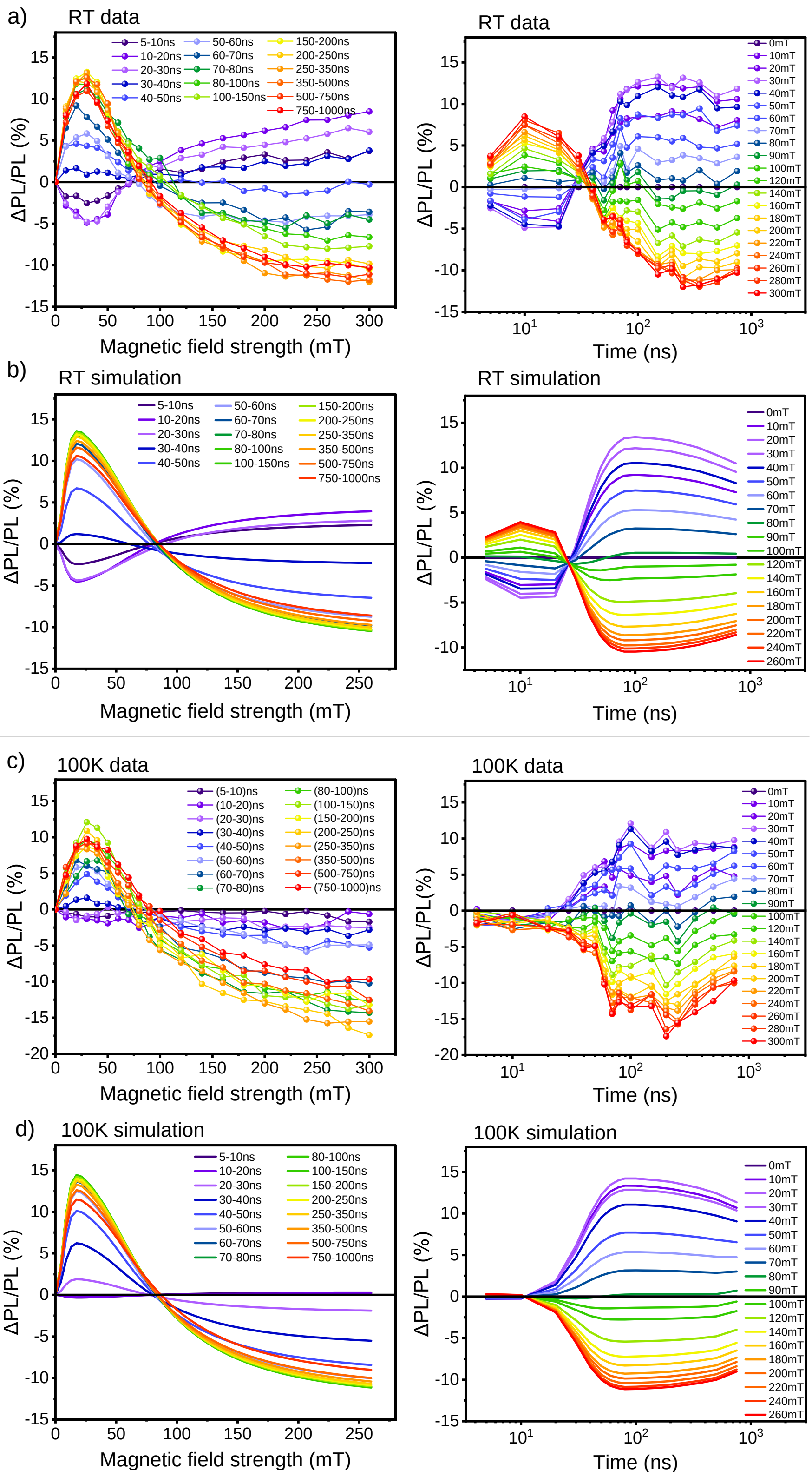

Figure S20: Comparison of experimental results (a,c) and simulations (b,d) of MFEs on the photoluminescence of the diF-TES-ADT drop-cast film measured at 532 nm across different delay times at (a,b) room temperature and (c,d) 100 K, respectively. The simulation and the data of the MFE at RT and 100 K are in reasonably good agreement.

7.3 Fluence-dependent simulation

To simulate the power-dependent MFE results at 100 K, the reported laser power, measured in µW, had to be converted to the exciton density, measured in cm^{-3}, before being applied to our updated code. Using the following equation, the values of the average exciton density N were calculated from the measured laser power P, where P was set to 11, 33, 100, 300, 900, and 2300 µW:

$$N = F_A(1 - F_S)R_P \frac{P\lambda}{f\pi r_x r_y dhc}$$

Here F_S and F_A represent the fractions of incident light scattered and absorbed by the sample respectively, which were determined from the UV–Vis transmission spectrum. For drop-cast diF-TES-ADT film, the sample thickness varies from 100 nm to 15 μm. Therefore, these fractions ranged from 0.2 to 0.45 for F_S, and from 0.21 to 0.34 for F_A. R_P is the ratio of measured power between the sample position and power meter position, which was determined to be 0.58 in our setup. The repetition rate of the laser, f, was set at 1 kHz for internally triggered power measurements. The radii of the excitation beam spot, r_x and r_y, are measured as 158.6 µm and 99.8 µm, respectively, using a CCD beam profiler from Thorlabs. The excitation wavelength is 532 nm, and h and c are Planck's constant and the speed of light, respectively. However, the potential excitation densities vary by approximately three orders of magnitude due to the non-uniform thickness of the drop-cast film. Accurately determining the excitation densities for this specific sample is somewhat challenging. Consequently, we'll be phenomenological and use the thin film's excitation densities in our simulation since we possess precise measurements of the film's thickness. Using these data to simulate our findings exhibits similar behaviour to the experimental fluence-dependent MFE data presented in the main text, as shown in Figure S21.

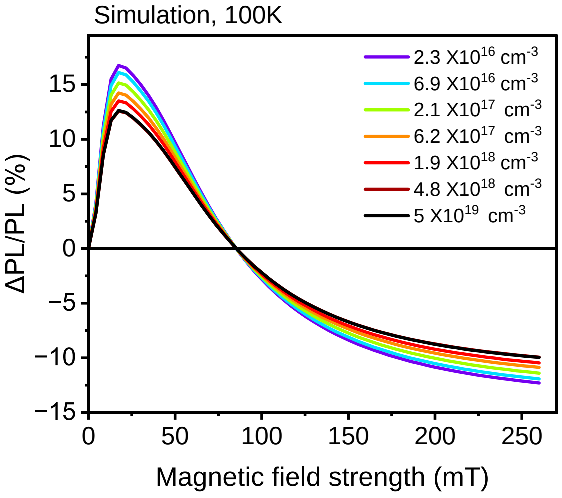

Figure S21: Simulation of the fluence- dependent magnetic field effect of diF-TES-ADT drop-cast film measured at 523 nm at 100 K. The simulation shows the drop in the TTA-MFE as the exciton density rises and broadly shows good agreement with the experimental MFE behavior.

Figure S21 shows the resulting fluence-dependent simulation exhibiting a drop in the TTA-MFE as the excitation density rises, which is consistent with the experimental data presented in the main text. It is important to observe that exceeding an exciton density of $5{\times}10^{18}$ results in signal saturation, whilst decreasing the exciton density below $2.3{\times}10^{16}$ yields an even greater signal. Thus, our data is likely within this range of exciton densities.

References

(S1) Hallani, R. K.; Thorley, K. J.; Mei, Y.; Parkin, S. R.; Jurchescu, O. D.; Anthony, J. E. Structural and electronic properties of crystalline, isomerically pure anthradithiophene derivatives. *Advanced Functional Materials* **2016**, *26*, 2341–2348.

(S2) Momma, K.; Izumi, F. VESTA: a three-dimensional visualization system for electronic and structural analysis. *Journal of Applied Crystallography* **2008**, *41*, 653–658.

(S3) Ashiotis, G.; Deschildre, A.; Nawaz, Z.; Wright, J. P.; Karkoulis, D.; Picca, F. E.; Kieffer, J. The fast azimuthal integration Python library: pyFAI. *Journal of Applied Crystallography* **2015**, *48*, 510–519.

(S4) Necas, D. Gwyddion: an open-source software for SPM data analysis. *Cent Eur J Phys* **2012**, *10*, 181.

(S5) Neese, F.; Wennmohs, F.; Becker, U.; Riplinger, C. The ORCA quantum chemistry program package. *J. Chem. Phys.* **2020**, *152*, 224108–1–18.

(S6) Weigend, F.; Ahlrichs, R. Balanced basis sets of split valence, triple zeta valence and quadruple zeta valence quality for H to Rn: Design and assessment of accuracy. *Phys. Chem. Chem. Phys.* **2005**, *7*, 3297–3305.

(S7) Weigend, F. Accurate Coulomb-fitting basis sets for H to Rn w. *Phys. Chem. Chem. Phys.* **2006**, *8*, 1057–1065.

(S8) Grimme, S.; Antony, J.; Ehrlich, S.; Krieg, H. A consistent and accurate ab initio parametrization of density functional dispersion correction (DFT-D) for the 94 elements H-Pu. *J. Chem. Phys.* **2010**, *132*, 154104–1–19.

(S9) Grimme, S.; Ehrlich, S.; Goerigk, L. Effect of the Damping Function in Dispersion Corrected Density Functional Theory. *J. Comput. Chem.* **2011**, *32*, 1456–1465.

(S10) Barone, V. In *Recent Advances in Density Functional Methods*; Chong, D. P., Ed.; World Scientific Publ. Co.: Singapore, 1995; Vol. 1; Chapter 8, pp 287–334.

(S11) Rega, N.; Cossi, M.; Barone, V. Development and validation of reliable quantum mechanical approaches for the study of free radicals in solution. *J. Chem. Phys.* **1996**, *105*, 11060–11067.

(S12) Huzinaga, S. Gaussian-Type Functions for Polyatomic Systems. I. *J. Chem. Phys.* **1965**, *42*, 1293–1302.

(S13) Kutzelnigg, W.; Fleischer, U.; Schindler, M. In *NMR Basic Principles and Progress Vol. 23*; Diehl, P., Fluck, E., Günther, H., Kosfeld, R., Seelig, J., Eds.; Springer: Heidelberg, 1990; p 165.

(S14) Bossanyi, D. G.; Matthiesen, M.; Wang, S.; Smith, J. A.; Kilbride, R. C.; Shipp, J. D.; Chekulaev, D.; Holland, E.; Anthony, J. E.; Zaumseil, J.; Musser, A. J.; Clark, J. Emissive spin-0 triplet-pairs are a direct product of triplet–triplet annihilation in pentacene single crystals and anthradithiophene films. *Nature Chemistry* **2021**, *13*, 163–171.

(S15) Yong, C. K.; Musser, A. J.; Bayliss, S. L.; Lukman, S.; Tamura, H.; Bubnova, O.; Hallani, R. K.; Meneau, A.; Resel, R.; Maruyama, M.; others The entangled triplet pair state in acene and heteroacene materials. *Nature communications* **2017**, *8*, 15953.

(S16) Matsuda, S.; Oyama, S.; Kobori, Y. Electron spin polarization generated by transport of singlet and quintet multiexcitons to spin-correlated triplet pairs during singlet fissions. *Chem. Sci.* **2020**, *11*, 2934–2942.

(S17) Okamoto, T.; Izawa, S.; Hiramoto, M.; Kobori, Y. Efficient Spin Interconversion by Molecular Conformation Dynamics of a Triplet Pair for Photon Up-Conversion in an Amorphous Solid. *J. Phys. Chem. Lett.* **2024**, *15*, 2966–2975.

(S18) Corvaja, C.; Franco, L.; Salikhov, K.; Voronkova, V. The first observation of electron spin polarization in the excited triplet states caused by the triplet-triplet annihilation. *Appl. Magn. Reson.* **2005**, *28*, 181–193.

(S19) Stoll, S.; Schweiger, A. EasySpin, a comprehensive software package for spectral simulation and analysis in EPR. *J. Magn. Reson.* **2006**, *178*, 42–55.

(S20) Tait, C. E.; Krzyaniak, M. D.; Stoll, S. Computational tools for the simulation and analysis of spin-polarized EPR spectra. *J. Magn. Reson.* **2023**, *349*, 107410–1–16.

(S21) Furrer, R.; Fujara, F.; Lange, C.; Stehlik, D.; Vieth, H. M.; Vollmann, W. Transient ESR nutation signals in excited aromatic triplet states. *Chem. Phys. Lett.* **1980**, *75*, 332–339.

(S22) Johnson, R.; Merrifield, R. Effects of magnetic fields on the mutual annihilation of triplet excitons in anthracene crystals. *Phys. Rev. B* **1970**, *1*, 896.

(S23) Piland, G. B.; Burdett, J. J.; Kurunthu, D.; Bardeen, C. J. Magnetic field effects on singlet fission and fluorescence decay dynamics in amorphous rubrene. *J. Phys. Chem. C* **2013**, *117*, 1224–1236.

(S24) Subramanian, S.; Park, S. K.; Parkin, S. R.; Podzorov, V.; Jackson, T. N.; Anthony, J. E. Chromophore fluorination enhances crystallization and stability of soluble anthradithiophene semiconductors. *J. Am. Chem. Soc.* **2008**, *130*, 2706–2707.
